# Prediction and Validation of a Protein’s Free Energy Surface Using Hydrogen Exchange and (Importantly) Its Denaturant Dependence

**DOI:** 10.1021/acs.jctc.1c00960

**Published:** 2021-12-22

**Authors:** Xiangda Peng, Michael Baxa, Nabil Faruk, Joseph R. Sachleben, Sebastian Pintscher, Isabelle A. Gagnon, Scott Houliston, Cheryl H. Arrowsmith, Karl F. Freed, Gabriel J. Rocklin, Tobin R. Sosnick

**Affiliations:** † Department of Biochemistry and Molecular Biology, University of Chicago, Chicago, Illinois 60637, United States; ‡ Graduate Program in Biophysical Sciences, University of Chicago, Chicago, Illinois 60637, United States; § Division of Biological Sciences, University of Chicago, Chicago, Illinois 60637, United States; ∥ Department of Molecular Biophysics, Faculty of Biochemistry, Biophysics and Biotechnology, Jagiellonian University, Kraków 30387, Poland; ⊥ Structural Genomics Consortium, University of Toronto, Toronto, Ontario M5G 1L7, Canada; # Department of Chemistry, University of Chicago, Chicago, Illinois 60637, United States; ∇ Department of Pharmacology & Center for Synthetic Biology, Northwestern University, Chicago, Illinois 60614, United States; ■ Princess Margaret Cancer Centre and Department of Medical Biophysics, University of Toronto, Toronto, Ontario M5G 2M9, Canada

## Abstract

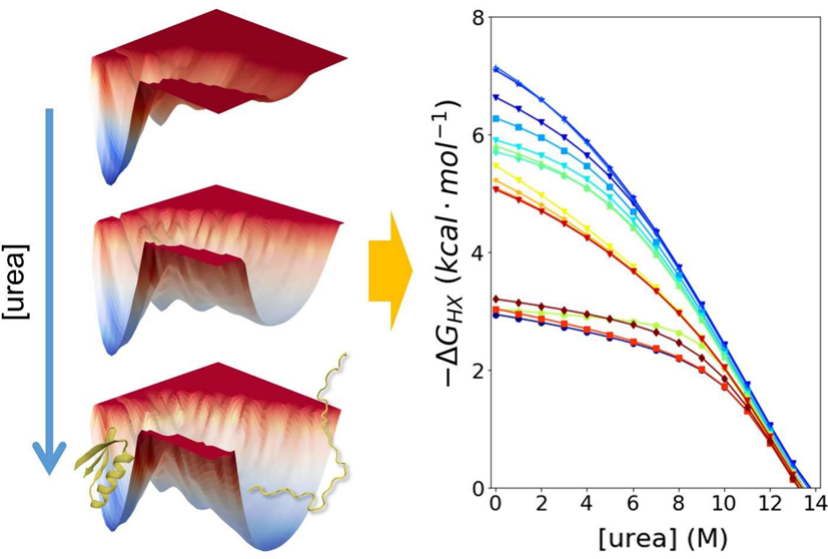

The denaturant dependence of hydrogen–deuterium exchange (HDX) is a powerful measurement to identify the breaking of individual H-bonds and map the free energy surface (FES) of a protein including the very rare states. Molecular dynamics (MD) can identify each partial unfolding event with atomic-level resolution. Hence, their combination provides a great opportunity to test the accuracy of simulations and to verify the interpretation of HDX data. For this comparison, we use *Upside*, our new and extremely fast MD package that is capable of folding proteins with an accuracy comparable to that of all-atom methods. The FESs of two naturally occurring and two designed proteins are so generated and compared to our NMR/HDX data. We find that *Upside*’s accuracy is considerably improved upon modifying the energy function using a new machine-learning procedure that trains for proper protein behavior including realistic denatured states in addition to stable native states. The resulting increase in cooperativity is critical for replicating the HDX data and protein stability, indicating that we have properly encoded the underlying physiochemical interactions into an MD package. We did observe some mismatch, however, underscoring the ongoing challenges faced by simulations in calculating accurate FESs. Nevertheless, our ensembles can identify the properties of the fluctuations that lead to HDX, whether they be small-, medium-, or large-scale openings, and can speak to the breadth of the native ensemble that has been a matter of debate.

## Introduction

Proteins populate high-energy states as determined by their free energy surfaces. These states are often relevant in folding, catalysis, binding, conformational selection, aggregation, and allostery.^1^ An ongoing challenge is to accurately calculate the free energy surface, including the generation of the Boltzmann ensemble of all major species. By providing the free energies for the breaking of individual hydrogen bonds, Δ*G*_HX_, HDX and its denaturant dependence is an excellent method to identify excited states and test the veracity of a simulated free energy surface.

**Figure 1:**
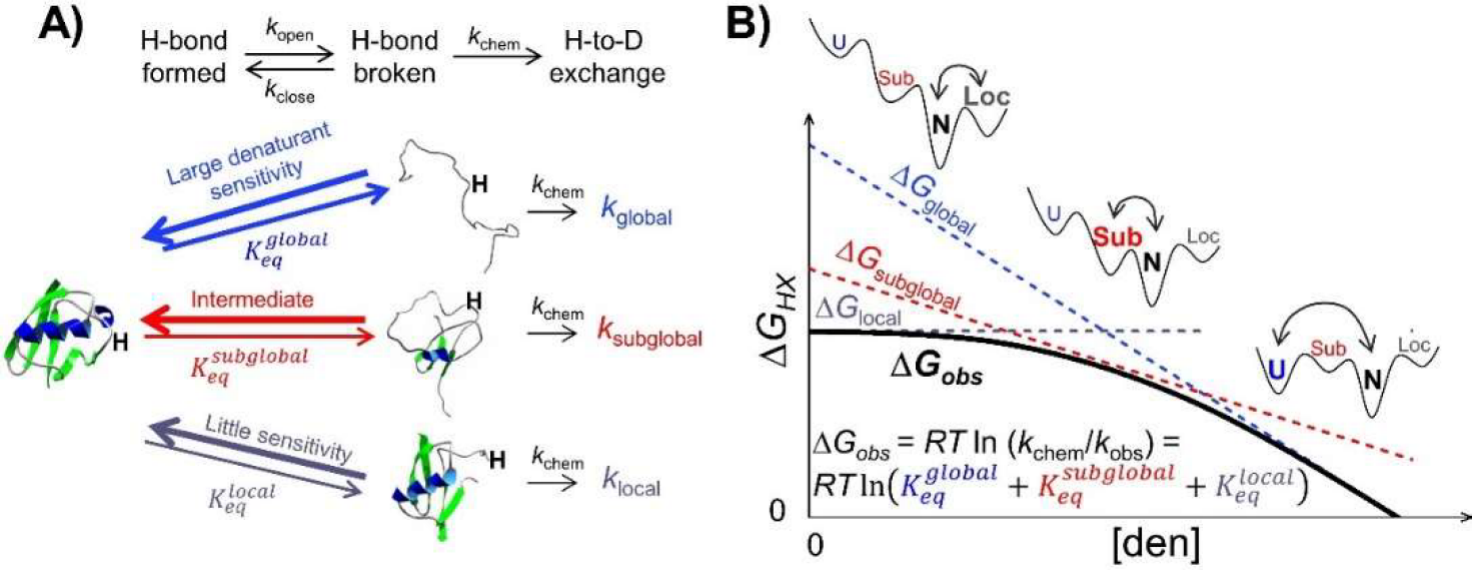
Denaturant dependence of HDX. (A) H-bonds are broken when the protein undergoes global, subglobal, and local openings that can be identified by their sensitivity to denaturant (*m*-values). (B) The observed exchange rate is the sum of the rates from each class of openings, global (U), subglobal (Sub), or local (Loc), which results in the observed denaturant dependence (black curved line). The free energy diagrams illustrate the shift in populations as the denaturant concentration is increased, and changes which state dominants the exchange process.

HDX occurs when an amide proton (NH) normally participating in an H-bond becomes exposed to solvent in a transient “open state” (Figure 1). A major advance in the interpretation of HDX data came about with the measurement of the denaturant dependence of exchange, which provides an indicator of the size of the opening event.^2,3^ For many proteins, HDX of the most stable H-bonds has a large denaturant dependence, and the exchange process corresponds to the global unfolding of the protein, for example, as measured by temperature or chemical denaturation.^4^ The other, less stable H-bonds have a reduced sensitivity to denaturant, indicating that the structural opening involves only a portion of the protein.

The size of the opening varies, ranging from only one or a few amides in “local” openings to larger “subglobal” openings to complete or “global” unfolding. Rather than the breaking of a specific H-bond, local opening events have been proposed to reflect population shifts within a broad native ensemble.^5,6^ For some proteins, subglobal openings likely represent unfolding intermediates where the disordered regions correspond to the unfolding of one or more helices and/or strands (“foldons”) that exchange in a concerted manner.^3^

The prediction of a free energy surface and its validation using HDX data is challenging. An implication of having one or more NHs exchanging with Δ*G*_HX_ matching the global stability Δ*G*_eq_, and its denaturant dependence, is that the amount of residual H-bonded structure must be minimal in the denatured state ensemble (DSE). Achieving such a high degree of folding cooperativity is difficult as it requires that the energy function strike the proper balance between the stabilizing interactions needed to fold the protein and the destabilizing protein–solvent interactions necessary to produce a DSE devoid of residual structure.^7,8^

All-atom molecular dynamics (MD) simulations in principle are well suited to generate Boltzmann ensembles for HDX calculations.^9−14^ Previously, however, we examined DESRES folding simulations for NuG2b, a small α/β protein, and found that the DSE possessed a near-native radius of gyration (*R*_g_) and native-like H-bonding levels. These properties are inconsistent with our HDX and small-angle scattering data.^15^ Generally, MD force fields have excess residual structure, which inhibits their ability to predict HDX, although improvements have been made.^7,8^

Here, we advance our near-atomic level MD *Upside* algorithm that can fold proteins with accuracy comparable to that of all-atom methods but in CPU-hours^16,17^ and test whether it can generate ensembles that can reproduce HDX data for four proteins: two Rosetta-designed proteins,^18^ mammalian ubiquitin, and a ubiquitin variant (L50E). To predict the larger openings and their denaturant dependence, we find it necessary to train our force field in a manner that reduces the amount of residual structure in the DSE and increases folding cooperativity. Although the agreement between our simulations and the HDX data is laudable, areas for improvement include increasing cooperativity and reducing the number of spurious small-scale openings. In general, our study provides a firmer foundation for testing simulations against HDX data, which should lead to improvements in our ability to simulate the free energy surface and interpret HDX data.

## Results

We first describe the *Upside* model and conduct dual-target contrastive divergence (ConDiv) training of our energy function using both the native state ensemble (NSE) and an unstructured DSE. This procedure is designed to balance the energy terms to decrease the amount of residual structure while still being able to fold proteins. Next, methods for comparing an MD trajectory to HDX data and its denaturant dependence are presented, followed by comparisons to data.

*Upside* is a near-atomic, implicit solvent model that conducts Langevin dynamics on just the three backbone N, C_α_, and C atoms, with the backbone conformation guided by neighbor- and residue-dependent (ϕ,ψ) torsion maps.^16,17^*Upside*’s speed in part arises from explicitly including only the three backbone atoms during the dynamics portion, but it uses the inferred positions of amide hydrogens, carbonyl oxygens, C_β_ atoms, and side chains during the force calculation. The side chains are represented by multiposition, amino acid-, and directional-dependent beads. After every Verlet integration time step, the bead position probabilities of all side chains are determined in a single global side chain packing step that produces the lowest side chain free energy. This step greatly reduces side chain friction, which, along with the lack of explicit solvent, explains much of the 10^3^–10^4^-fold speedup as compared to standard MD.

### Contrastive Divergence (ConDiv) Training

**Figure 2:**
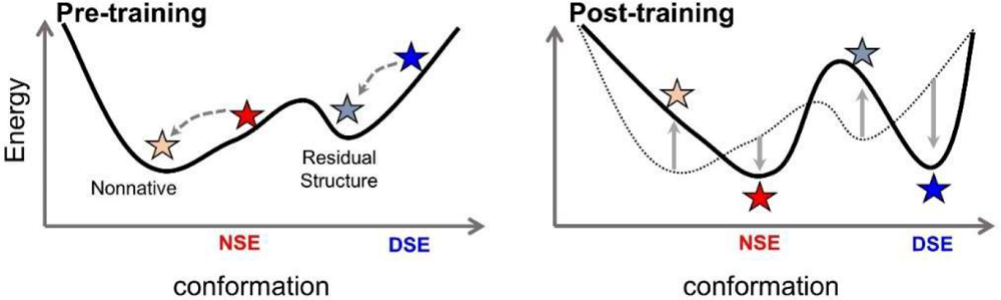
“Dual objective” contrastive divergence training. Left: Before training, simulations that start from the DSE (blue) or NSE (red) are unstable and relax to an ensemble with residual structure or misfolded states, respectively. Right: Force field parameters are iteratively updated to increase the native stability while reducing the amount of residual structure in the DSE. Ideally, new simulations that start in the DSE and NSE retain their respective conformational ensembles (dotted gray energy surface becomes solid black).

Critical to *Upside*’s success is the development of a force field having the proper balancing of energy terms. Balancing is achieved by simultaneously training essentially all parameters using our version of the machine learning ConDiv method.^17^ Here, one considers two ensembles, the first restrained to be near the native structure and the second that is free to diffuse away during a simulation. For a perfect energy function, the unrestrained ensemble will remain close to the native ensemble. However, differences will arise with an imperfect function, which can be reduced by changing the strength of the energy terms to preferentially stabilize the native conformers as compared to the unconstrained ensemble. For example, if too many H-bonds form in the unconstrained ensemble, the H-bond energy is reduced, and the training simulations are repeated. This iterative procedure of updating the energy terms to preferentially stabilize the NSE continues until no energy parameter can be updated to produce a better NSE (Figure 2). To avoid overtraining, this procedure is run for 456 proteins in batches of 19 to produce our 2018 energy function, “FF1”.

An improved FF2 version is obtained in the present study by adding an explicit backbone desolvation term, a better side chain burial calculation to include the chemistry of the side chain’s environment, and modifying the H-bond energy to be secondary structure-dependent (varies by 10%, Supporting Information Improving the Energy Function). These modifications follow FF1’s overall philosophy of using as few physically based energy terms as possible while still maintaining accuracy (Supporting Information Improving the Energy Function).

At temperatures above the melting temperature (*T*_m_), the DSE with FF1 has minimal H-bonded structure, but below the *T*_m_, some residual structure remains in the DSE (Figure S6). To reduce this structure and increase folding cooperativity, we developed a ConDiv procedure where training is conducted on both the DSE and the NSE simultaneously. The goal is to achieve the delicate balance between reducing the amount of residual structure in the DSE while still being able to fold proteins. A DSE training ensemble was generated using expanded conformations from simulations run at ∼*T*_m_ (Figures 2 and S3, Supporting Information Parameterization by ConDiv). The target ensemble for the DSE was taken to be a self-avoiding random walk (SARW). This dual-target ConDiv training procedure was used to obtain FF2 (Supporting Information Parameterization by ConDiv).

**Figure 3:**
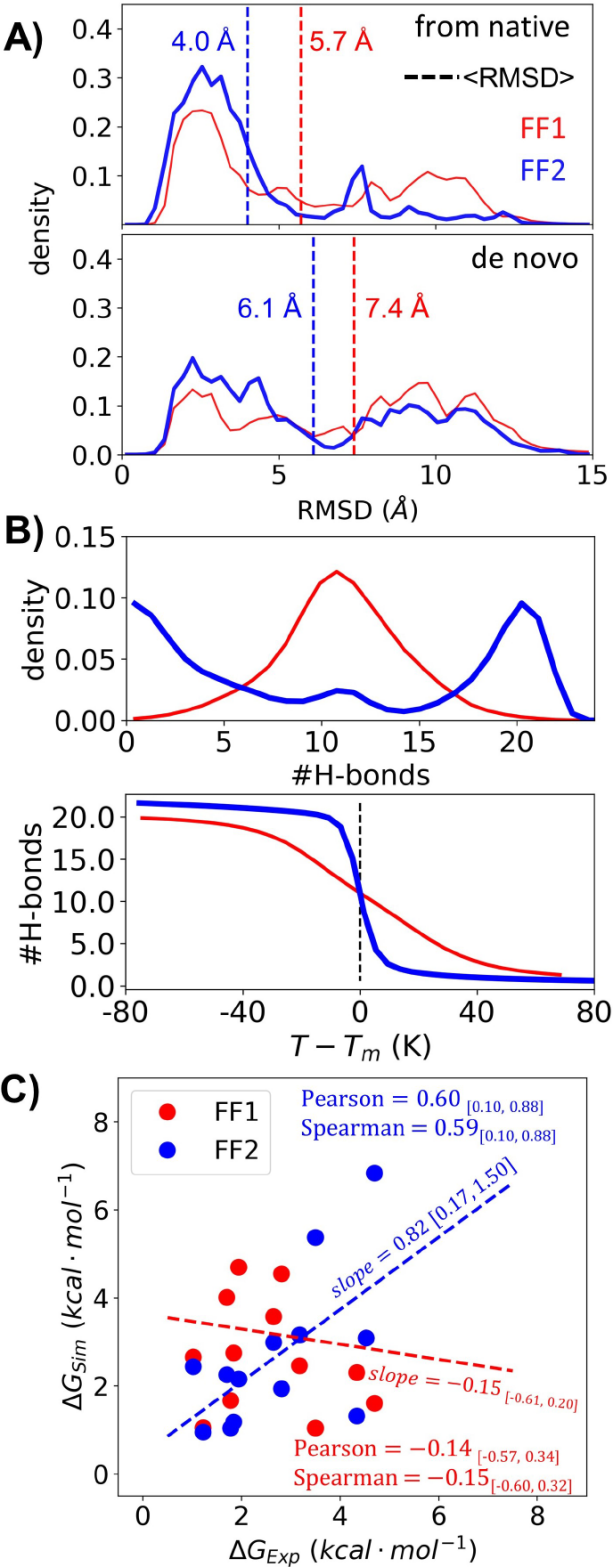
Improved performance with force field FF2. (A) C_α_-RMSD distribution of the validation set starting from either a native or an unfolded conformation (average values indicated with dashed lines). (B) H-bond distribution at the *T*_m_ for a set of designed mini-proteins and the corresponding melting curves. (C) Predicted and experimental Δ*G* values at 298 K for the mini-proteins using the original FF1 and the new FF2 energy function. The correlation coefficients and the slope of the best fit are listed with a 90% confidence interval.

The performance of FF2 was evaluated in terms of structural accuracy for *de novo* folding, the ability to remain in the native well, the degree of folding cooperativity, and the overall thermodynamic stability. The ability of FF2 to both fold proteins *de novo* and maintain the native state as a stable minimum was noticeably improved for a validation set of 16 proteins. For *de novo* folding with FF1 and FF2, the average TM-scores^19^ were 0.37 (⟨C_α_-RMSD⟩ = 7.4 Å) and 0.42 (⟨C_α_-RMSD⟩ = 6.1 Å), respectively. TM-score is a similarity metric that is independent of protein size and reflects the fraction of the predicted structure that can be aligned to the native or some other reference structure within a certain distance cutoff (e.g., 5 Å).^19^ Likewise, for trajectories starting from the native state, the TM-scores of the excursions within the native basin were 0.45 and 0.55 (RMSD values of 5.7 and 4.0 Å), respectively (Figures 3A, S4, and S5). The improvement in the ability to remain near the native state is especially significant as the trajectories that begin in the native state are those that are used to calculate HDX data.

To examine the importance of our dual-target ConDiv procedure, we created FF1+, a version that contains the extra energy terms found in FF2 but lacks the dual-target training protocol. A comparison of FF1+ to FF1 indicates that the new energy terms did help increase folding cooperativity (Figures 3, S6, and S7), but they did not significantly improve our estimation of global stability (Figures S8 and S9). Hence, our dual-target ConDiv training procedure is a key factor for the improvement of FF2. Effectively, having a disordered DSE serves as a regularizer that produces the minimum interaction energy that can still generate both a stable NSE and a disordered DSE.

The extent of folding cooperativity in FF2 was assessed by comparing the fraction of H-bonds formed in the conformations that lie outside the NSE to the fraction that are in the NSE at the *T*_m_. After dual-target training, the H-bond distributions show increased bimodal character, and the ratio of the non-NSE H-bond fraction to the NSE H-bond fraction decreased from 38–50% to 20–31% for two Rosetta-designed mini-proteins, mammalian ubiquitin and an L50E variant (Figures 3B, S6, and S7). This reduction in residual H-bond levels should improve our ability to identify large opening events using HDX.

To make a direct comparison to the experimental data, our first step was to calibrate the *Upside* temperature scale using the measured stability of 13 Rosetta-designed mini-proteins (Table S1) that *Upside* can reversibly fold to near-native resolution, C_α_-RMSD ≈ 1.2–5.0 Å (average 2.3 Å) at 298 K.^18^ For each protein, temperature replica exchange molecular dynamics (T-REMD) simulations^20,21^ were run to generate melting curves that were fit assuming a two-state U-to-N model (Figure S8). The single *Upside* simulation temperature that best reproduced the experimental stabilities for the 13 proteins was defined to be 298 K (Supporting Information Temperature Calibration).

For this set, *Upside*’s estimation of global stability differs from experiment by an average of 1.3 kcal·mol^–1^ (⟨Δ*T*_m_⟩ = 7 K). The stability predicted by FF2 showed an improved correlation with the experimental values, having a Pearson correlation coefficient of 0.60 and a slope of 0.82, as compared to values of −0.14 and −0.15 for FF1 (Figures 3C and S9). This improvement is notable as the changes in FF2 were not explicitly intended to improve *Upside*’s ability to calculate stability.

### Free Energy Surface of Two Designed Mini-Proteins

**Figure 4:**
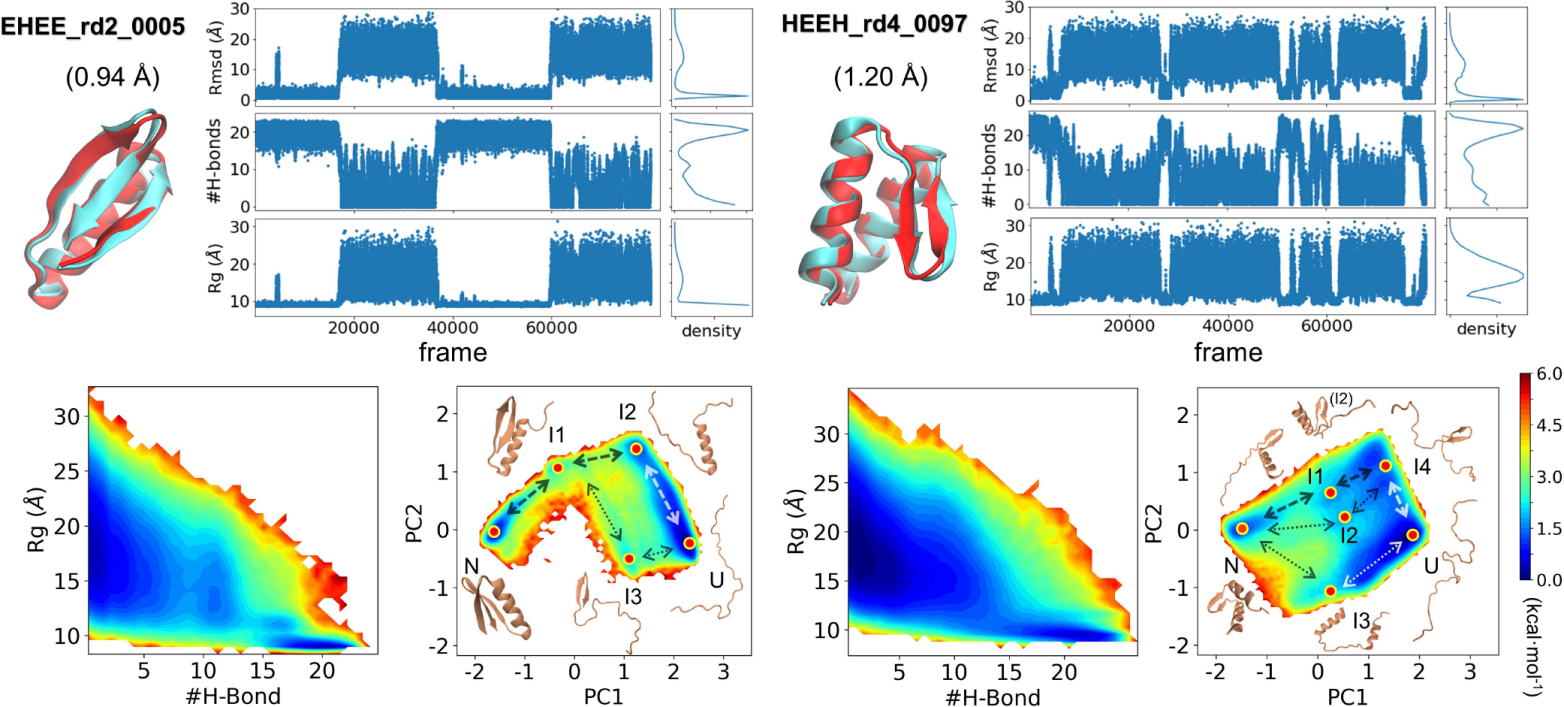
Reversible folding of EHEE_rd2_0005 (left) and HEEH_rd4_0097 (right). Predicted (cyan) and Rosetta-designed targets (red) are shown along with reversible folding trajectories, corresponding histograms, and heat maps of the FES at the *T*_m_ (320 K for EHEE_rd2_0005, 317 K for HEEH_rd4_0097). The FES is shown using two different sets of axes: (1) *R*_g_ and the number of H-bonds and (2) the first two components in the PCA. Representative pathways and structures are shown, which highlight the diverse folding routes.

We ran *Upside* simulations for two Rosetta-designed α/β mini-proteins, termed EHEE_rd2_0005 (40 residues) and HEEH_rd4_0097 (43 residues).^18^ EHEE_rd2_0005 has a three-stranded β sheet with a helix inserted between the amino-terminal strand and the carboxy-terminal hairpin. HEEH_rd4_0097 has a central hairpin flanked by two helices. Reversible folding/unfolding behavior is observed for both proteins with the NSE having an RMSD ≈ 1 Å from the designed targets (Figure 4) and 1.1–1.7 Å to the 20 NMR structures (PDB: 5UYO)^18^ for HEEH_rd4_0097.

To obtain the free energy surface, we performed T-REMD simulations and combined the different replicas using the multistate Bennett acceptance ratio (MBAR) method.^22^ For both proteins, the free energy surface contains two distinct energy wells for the NSE and DSE, the latter of which is largely devoid of H-bonds (Figures 4 and S13). Principal component analysis (PCA) was conducted using the C_α_–C_α_ contact matrix as the coordinates (C_α_–C_α_ distance < 10 Å). For EHEE_rd2_0005, two native-like intermediates appear that contain either the helix (I2) or the helix plus hairpin (I1). A minor folding pathway exists, which contains a lowly populated intermediate having only the carboxy hairpin (I3) at a population of only 5 × 10^–4^ relative to I2 at 287 K (Figures 4 and S14). For HEEH_rd4_0097, there are two minor depressions in the free energy surface representing the loss of one or both helices, while an even lower populated species is observed lacking the internal hairpin (Figure 4). The PCA map suggests three reversible pathways, but none is dominant (Figure S15). These simulated ensembles were used to calculate HDX patterns.

### HDX Calculations

HDX occurs when an H-bond is broken in a transient open state, and the NH is exposed to solvent:^23^
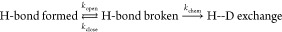
The observed HDX rate (*k*_obs_) can be expressed as
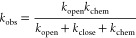
The relative slowing of *k*_obs_ as referenced to the intrinsic chemical exchange rate, *k*_chem_, is termed the protection factor (*PF = k*_chem_/*k*_obs_). Under the typical “EX2” condition where the closing rate, *k*_close_, of the H-bond is much faster than *k*_chem_, the PF is directly related to the equilibrium stability according to
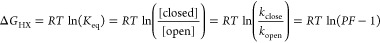
(See the section Testing for EX2 Behavior.) In our ensembles, exchange competent NHs are identified using Halle et al.’s criteria that the H-bond must be broken and the NH be coordinated to at least two nearby waters.^9^ These criteria are adapted to our implicit solvent simulations using our backbone burial level (*BL*^H^) term and H-bond score. This score is bimodal, being near 0 (broken) or 1 (made) during the simulations (Figure S11A). The burial level of the NH (*BL*^H^) comes from two contributions (Figure S11B), the heavy atoms on the backbone (*BL*_bb_^H^) and the side chain bead (*BL*_sc_^H^):
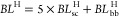
The value of 5 is used to increase the side chain score relative to that of the three backbone atoms, amide proton, and oxygen as the side chain bead is a single interaction center and hence under-represents the volume occupied by the side chain (Figure S11C). An NH is considered protected (*PS*_*i*_ = 1) when it is either H-bonded or buried with *BL*_*i*_^H^ > 5; otherwise, *PS*_*i*_ = 0. For a simulation, the free energy for each NH is calculated according to
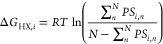
where *n* is the frame index and *N* is the number of frames.

### Calculating the Denaturant Dependence of HDX

The analysis of the denaturant dependence of Δ*G*_HX_ parallels that used in equilibrium measurements where the global stability is linearly dependent on the denaturant concentration, Δ*G* = Δ*G*_0_ – *m*_0_[den]. For global unfolding, the *m*_0_ value is proportional to the denaturant-sensitive surface area exposed upon unfolding of the protein.^24^ Similarly, for HDX, the slope of Δ*G*_HX_ versus [den] reflects the size of the lowest energy opening where NH is in an exchange competent state. Such openings generally occur through global, subglobal, and local openings^3,25−33^ (Figure 1). The HDX for these three classes has a large, medium, or near-zero sensitivity to denaturant, respectively.

A complicating aspect with HDX is that any given NH can exchange through multiple processes with fluxes that depend on the relative energy of each process, which can change with denaturant concentration. For example, for an NH that can be opened in each of the three classes, the net HDX rate is given by
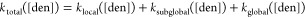
This NH may start exchanging through a local opening if Δ*G*_local_ < Δ*G*_subglobal_, Δ*G*_global_ in the absence of denaturant. With added denaturant, larger openings are preferentially promoted as they are more sensitive to denaturant. As a result, the NH that started exchanging via a local opening will transition to exchanging through openings having a stronger denaturant dependence with increasing denaturant once these opening events become the lowest free energy states. Likewise, a subglobal exchange process may in turn be overtaken by the global opening once the global opening becomes the open state with the lowest free energy. The transition from one class of opening to another provides a stringent test of a model’s ability to generate an accurate Boltzmann ensemble.

To include denaturant in the simulations, it is assumed to destabilize individual conformations proportional to the number of the protected backbone NHs (*N*_closed_^HB^) according to
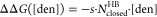
where *s* is a scale factor that is selected to reproduce the slope of the experimental denaturant dependence for each protein (Table S4). The value of ΔΔ*G*([den]) for each conformation is used to reweight its population at each simulated denaturant concentration. The use of NH protection as a proxy for the effects of urea is supported by a strong correlation between the number of H-bonds and exposed surface area in our simulations (*R*^2^ = 0.97, Figure S12) as well as HDX and transfer studies that indicate that backbone exposure relates to denaturant sensitivity for urea.^34,35^

### Comparison to Experiment

**Figure 5:**
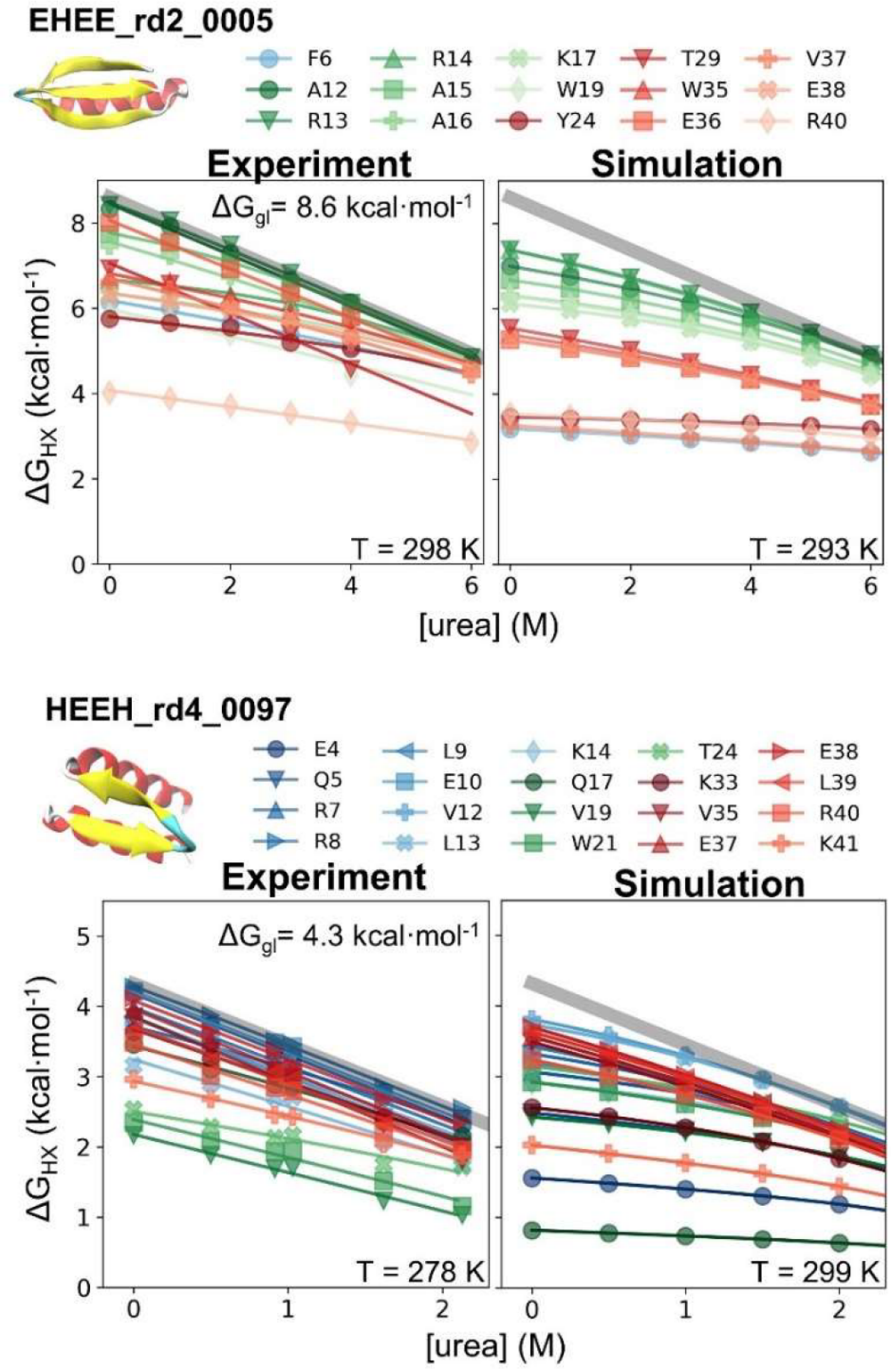
HDX and its denaturant dependence for EHEE_rd2_0005 and HEEH_rd4_0097. Only residues with experimentally determined Δ*G* values are shown.

HDX data were acquired for EHEE_rd2_0005 and HEEH_rd4_0097, respectively, at 298 K, pD_read_ 7.1 and 278 K, pD_read_ 4.6, where the most stable site on each protein has a stability of 8.6 and 4.3 kcal·mol^–1^. To focus on comparing FESs, we chose to compare the simulations at temperatures of 293 and 299 K where the simulated stabilities best match the experimental values for EHEE_rd2_0005 or HEEH_rd4_0097, respectively (Figure 5; Supporting Information Experimental Data Fitting, with comparisons at the experimental temperatures presented in Figure S16).

**Figure 6:**
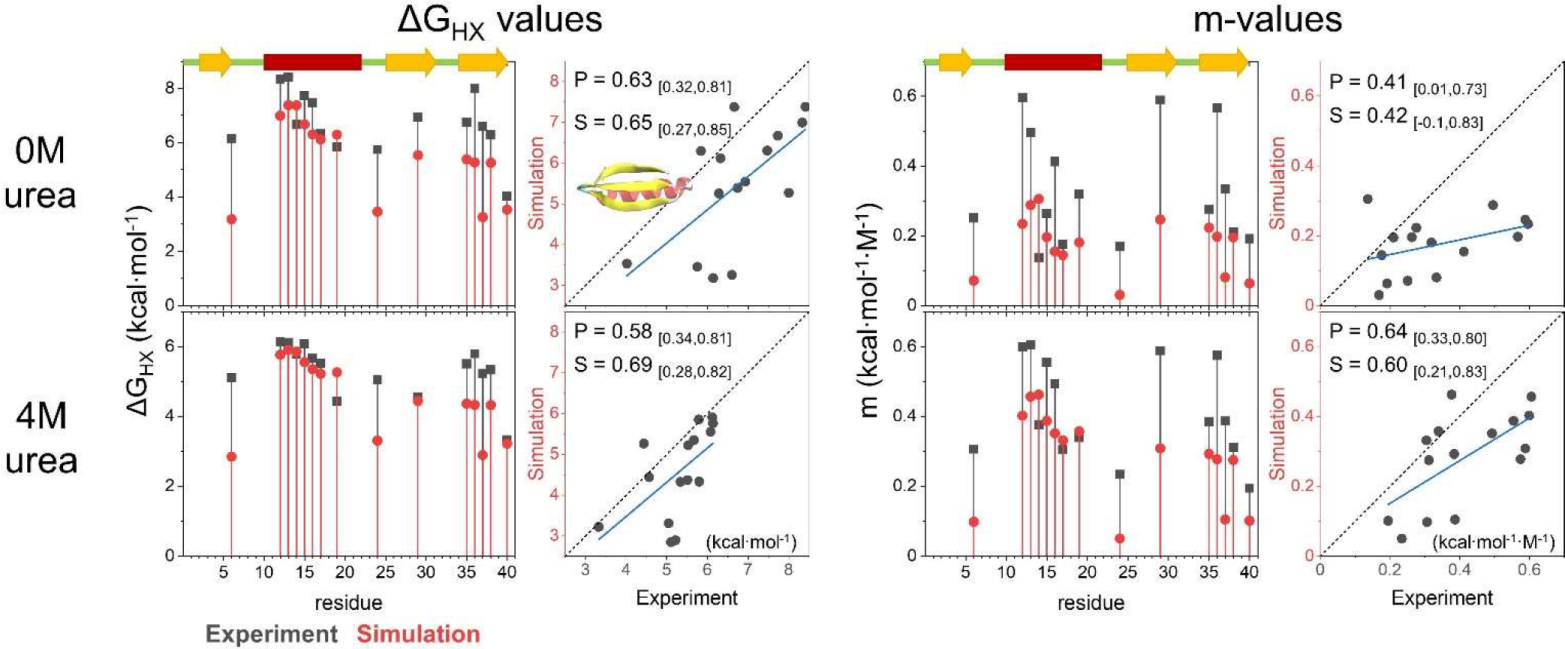
Comparison of the experimental and simulated Δ*G*_HX_ and *m*-values for EHEE_rd2_0005. Values from experiment and simulation are compared in the absence and presence of denaturant. The corresponding secondary structure is shown on the upper left (strands in yellow, helices in red). The correlation is shown on the right in each panel. The blue line is the best fit line. The Pearson (*P*) and Spearman (*S*) correlation coefficients and the 90% confidence interval (in the parentheses) are provided.

**Figure 7:**
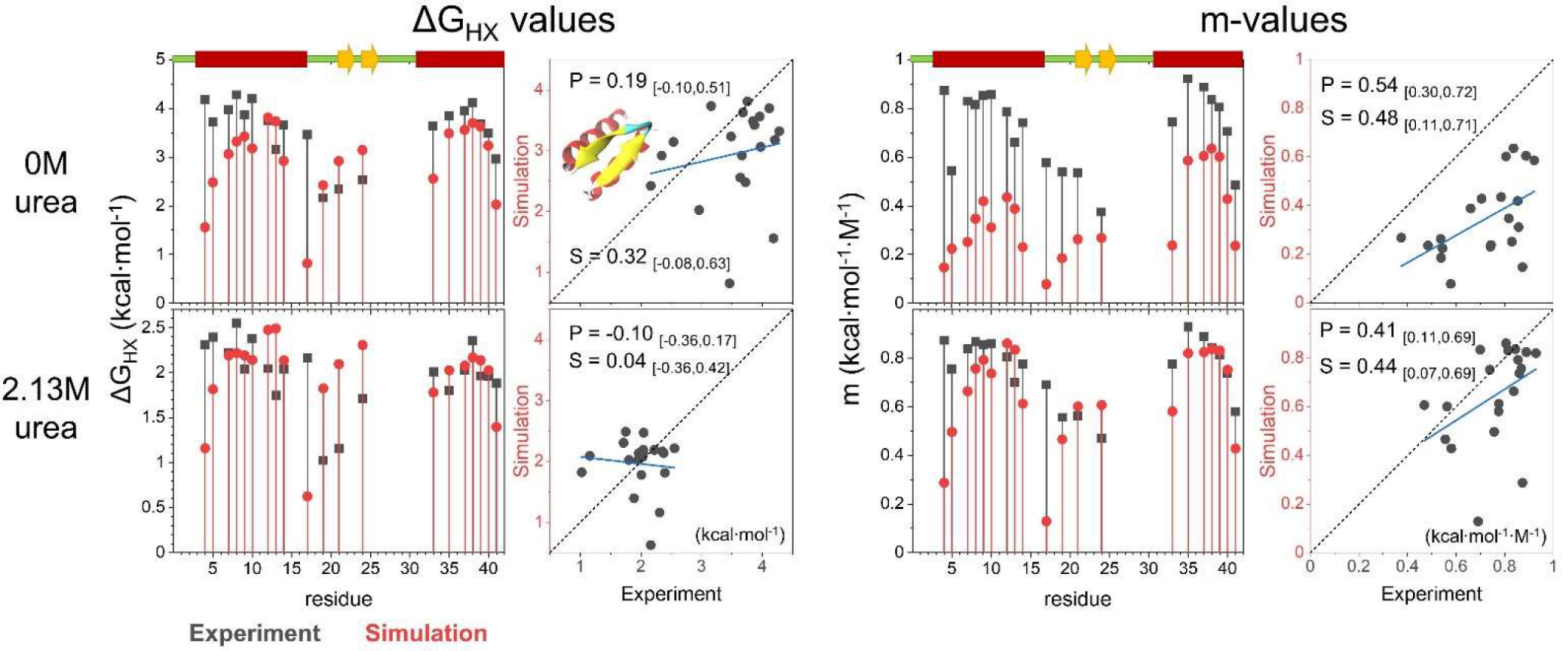
Comparison of the experimental and simulated Δ*G*_HX_ and *m*-values for HEEH_rd4_0097.

The pattern of site-resolved Δ*G*_HX_ and *m*-values from simulations is similar to that of their experimental counterparts (Figures 6, 7, and Table S14). As compared to the original FF1, the dual-target training of FF2 improved the prediction of HDX (Figure S16). The RMSE of FF2 to experimental Δ*G*_HX_ values is smaller by 2.5 and 0.3 kcal·mol^–1^ for EHEE_rd2_0005 and HEEH_rd4_0097, respectively (Figure S17). The RMSE of the *m*-value is also lowered by 0.05 to 0.1 kcal·mol^–1^·M^–1^, which indicates that folding is more cooperative with FF2.

Both proteins have openings with mild to large denaturant dependence, although for the simulations with FF2, neither protein has any sites that exchange only via globally unfolding. The highest Δ*G*_HX_ values for EHEE_rd2_0005 and HEEH_rd4_0097 from simulation fall short by 1.2 and 0.5 kcal mol^–1^, respectively.

An examination of the simulated free energy surface provides an explanation for why there are no global exchanging sites (Figure 4). For EHEE_rd2_0005, an intermediate exists on the major folding route that contains just the helix. While the helix potentially could then exchange through global unfolding from this state, the helix has another exchange route with an energy just below the global stability where only the β-hairpin remains intact. In fact, every secondary structure in EHEE_rd2_0005 can exchange prior to global unfolding on some pathway between the native and unfolded state. Likewise, for HEEH_rd4_0097, the helices and the hairpin can unfold independently. As a result, all sites can more readily exchange through a subglobal opening than through global unfolding.

Hence, *Upside* has not quite achieved the folding cooperativity under native conditions needed to reproduce the all-or-none behavior seen in the many proteins having sites that only exchange through global unfolding. At elevated urea concentrations, however, *Upside*’s performance is improved as the simulated *m*-values of most residues approach *m*_global_, and the individual Δ*G*_HX_ traces merge into the global unfolding “isotherm” seen experimentally and computationally.

### HDX Calculations for Ubiquitin and an L50E Mutant

We next studied ubiquitin (Ub, 76 residues) and a 5–6 kcal·mol^–1^ destabilized L50E variant.^32^ The substituted glutamic acid is located on the β5 strand and points toward the hydrophobic core of the protein. Rather than undergoing an energetically costly p*K*_a_ shift to the neutral Glu° form and remaining folded, the β5 strand unfolds to solvate the Glu^–^ state.^32^ As a result of this “vivisection” strategy, the ground state of the L50E mutant lacks a folded β5 strand.

*Upside* is able to fold Ub and the L50E variant to within 4.0 Å under stabilizing conditions (280 K) beginning from an unstructured state. When starting from the native state, the native structure is maintained to within 3.5 Å (Figures S18 and S19). However, we have difficulty observing reversible refolding in a single CPU-week due to the presence of long-lived misfolded species (Figure S20). These kinetically trapped states prevent refolding for trajectories that have passed from the native state to the unfolded state. Given that there is no reversibility, the free energy surface cannot be properly sampled as is the case for the mini-proteins.

**Figure 8:**
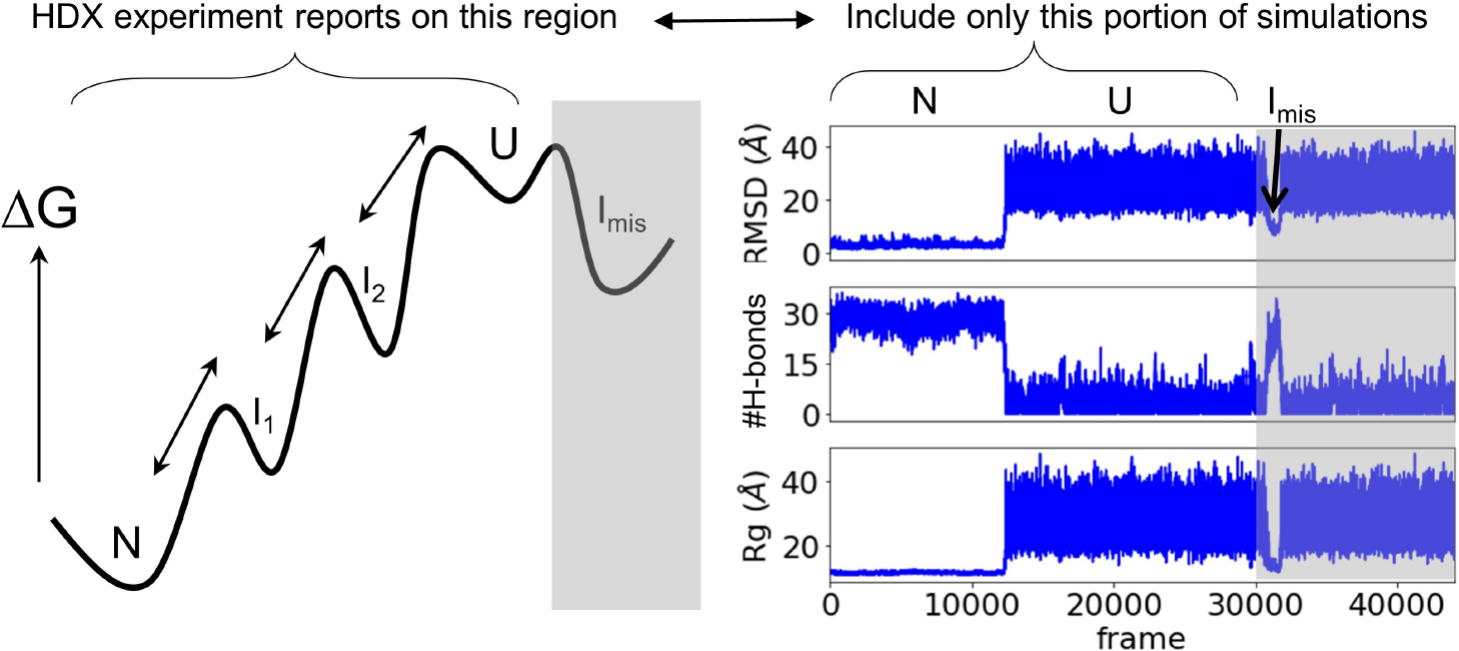
Proposed strategy for calculating HDX patterns when folding is irreversible. HDX is calculated using only the portion of the trajectories that go from the native to the unfolded state (U), prior to misfolding (gray regions).

To circumvent this issue, we propose that one can still obtain a reasonable conformational ensemble to predict HDX by including only the structures present on the free energy surface lying between the NSE and DSE (Figures 8 and S22). In this procedure, which mimics the actual HDX experiment as the protein starts from the native state, trajectories beginning in the native state are allowed to continue until the protein unfolds and then tries to refold. From this point forward, no more conformations from this trajectory are included in the HDX calculation. Because the DSE is under-sampled, this method may overestimate the native stability. However, if the protein adequately samples the region of the FES between the NSE and the DSE (Figure S21), then Δ*G*_HX_, which is referenced to the native state, should be correct for the states that lie between the NSE and DSE.

**Figure 9:**
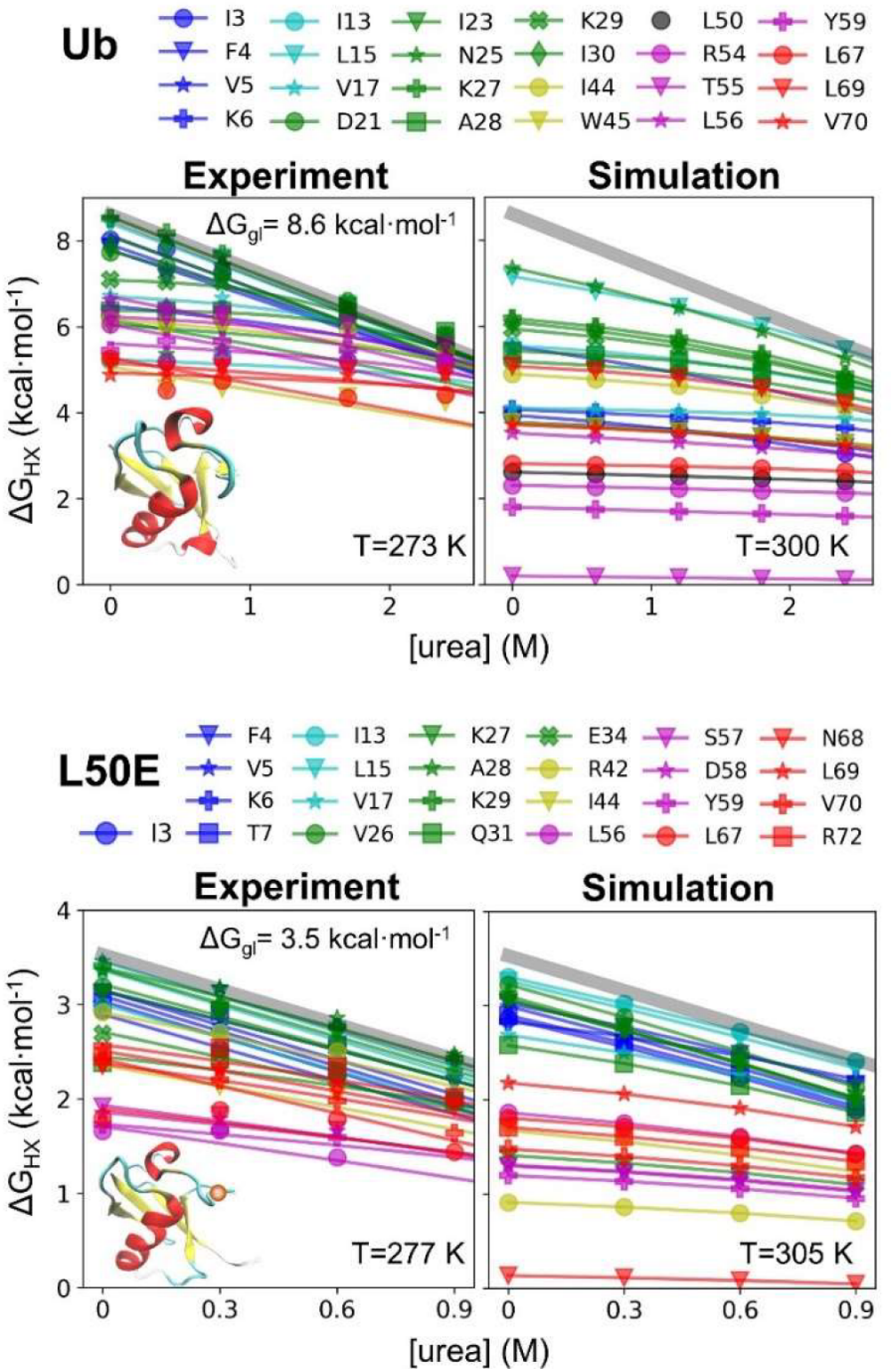
HDX data for Ub and the destabilized L50E variant.

We compared the results of this strategy to new HDX data on Ub acquired at 273 K, pD_read_ 7.6 and published data on L50E (277 K, pD_read_ 7.5).^32^ According to data for the sites with the largest Δ*G*_HX_ and *m*-values, the global stabilities are 8.6 and 4.7 kcal·mol^–1^ for the Ub and the variant, respectively (Figure 9). For purposes of comparing the HDX patterns, we selected simulation temperatures of 300 and 305 K to match the experimental stabilities (simulations at the experimental temperatures are shown in Figure S25).

As with the designed proteins, the simulated Ub and L50E ensembles did not have any sites that exchanged solely by global unfolding in the absence of urea, although L50E came close. The most stable NHs in the simulations were 1.3 and 0.2 kcal·mol^–1^ less stable than their experimental counterparts. Consistent with the experiment, the simulations found that the amino-terminal hairpin and helix are more stable than the carboxy-half of the protein, which largely exchanged through smaller-scale openings (lower *m*-values). As the denaturant increased, the *m*-values increased as larger openings were preferentially stabilized by denaturant.

**Figure 10:**
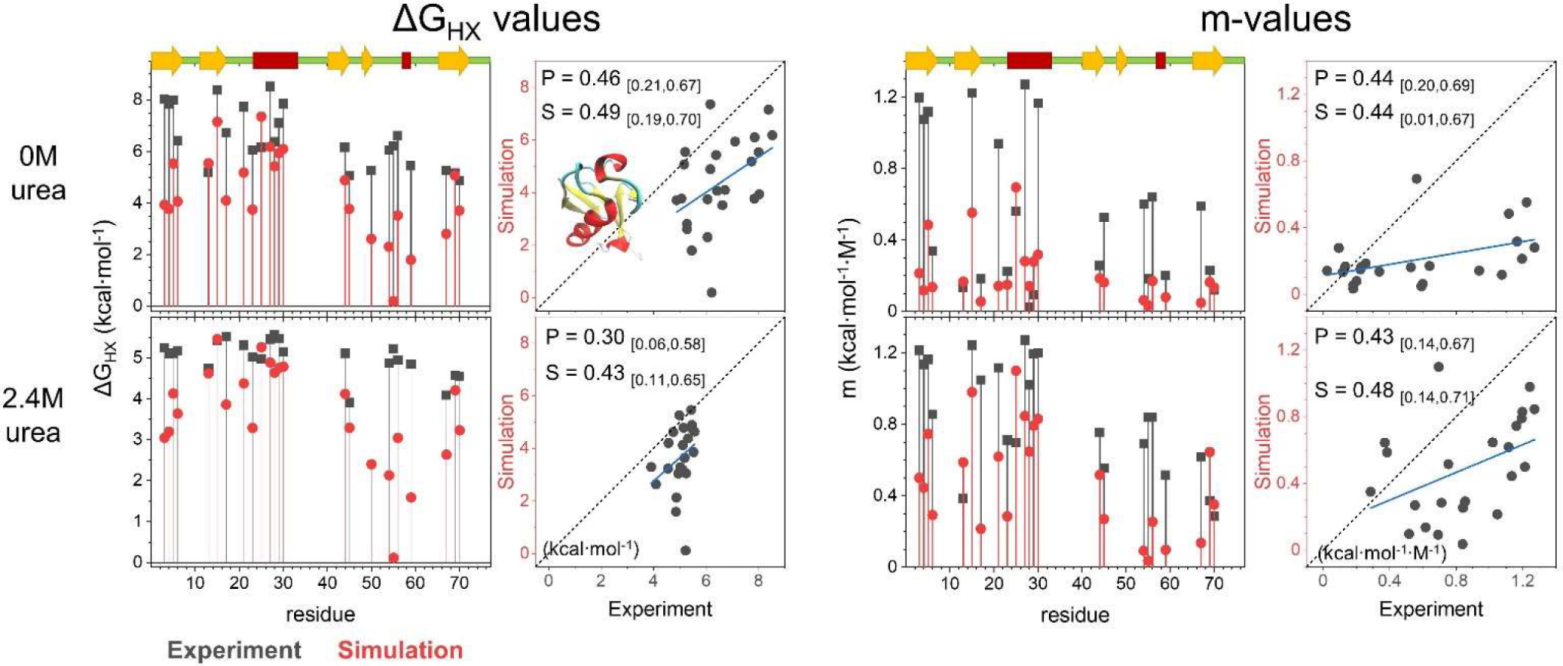
Comparison of the experimental and simulated Δ*G*_HX_ and *m*-values for Ub.

**Figure 11:**
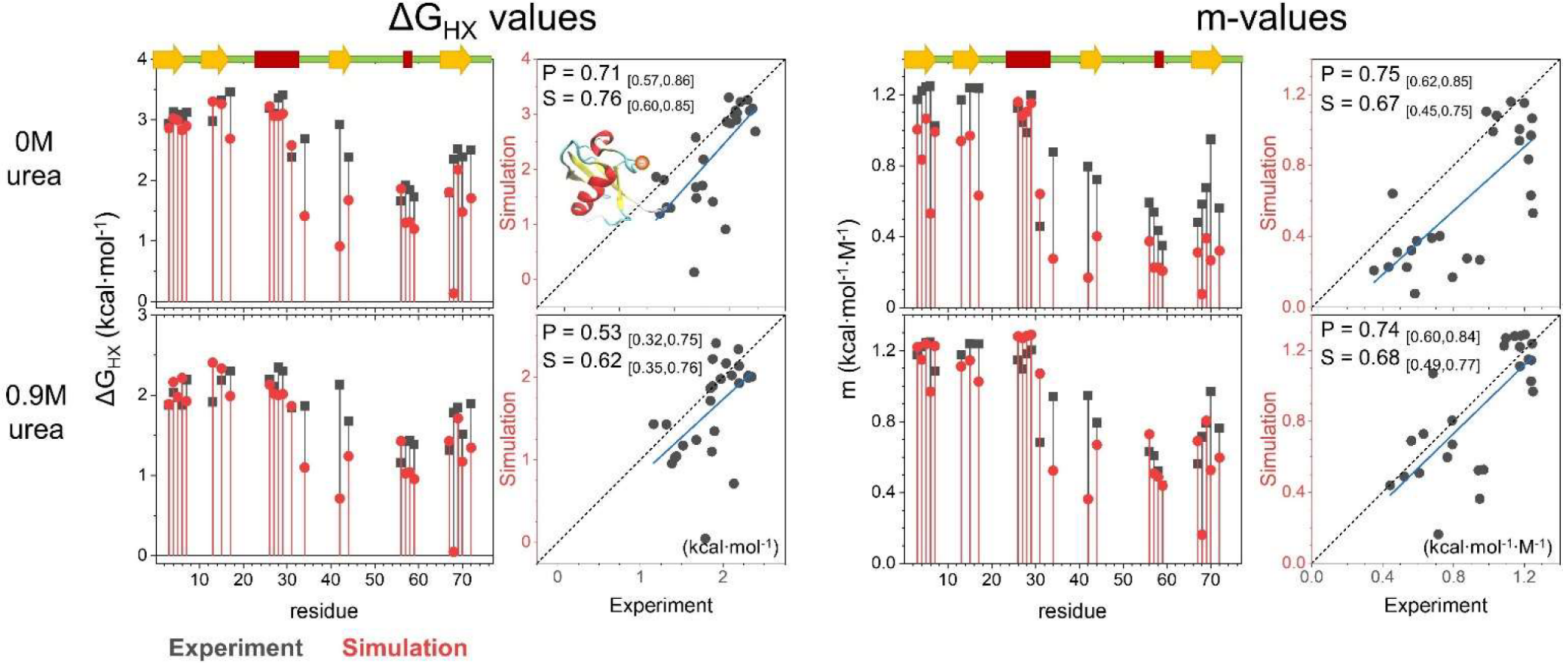
Comparison of the experimental and simulated Δ*G*_HX_ and *m*-values for L50E.

The overall HDX pattern and its denaturant dependence were much better predicted for the L50E variant (Figures 10, 11, and Table S14). This difference is due in part to the 4 kcal·mol^–1^ decrease in the energy gap between the native and the fully unfolded state for the variant. For larger energy gaps, there is an increased probability that a spurious small-scale opening event occurs in the simulations that can be the dominant exchange route and worsen the agreement with the HDX data. Examples of such fluctuations are β-strand register shifts of 1 or 2 residues (Figure S23).

The free energy surfaces for Ub and the variant were calculated from the same portions of the trajectories used to calculate the HDX pattern. The surfaces contain two defined wells corresponding to the NSE and DSE (Figure S23). The PCA map has two additional wells near PC1=2 representing states with the β2−β3 strand pairing having different registers. There are various states between the NSE and DSE, and the projection of the trajectory onto the PC1 and PC2 axes finds multiple pathways with the amino-terminal hairpin and α-helix being the most stable structures, consistent with the HDX data (Figure S24).

### Interpretation of Small *m*-Values

The *m*-value is the denaturant dependence of an H-bond’s free energy, reflecting the difference in the amount of exposed denaturant-sensitive area between the ensemble having the H-bond broken and the ensemble having the bond formed. We approximate the *m*-value as the difference in the total number of H-bonds formed in the closed and open states, that is, *m* ∝ *N*_closed_^HB^ – *N*_open_^HB^.

Traditionally, a small *m*-value is interpreted as an opening event involving only one or a few H-bonds in an otherwise very native-like conformation, that is, *N*_open_^HB^ ≈ *N*_total_^HB^ – 1. In principle, however, a small *m*-value could still occur in a broad native well having multiple H-bonds broken at any given time, so long as *N*_closed_^HB^ – *N*_open_^HB^ is small for the specific site under consideration.^5,6^

**Figure 12:**
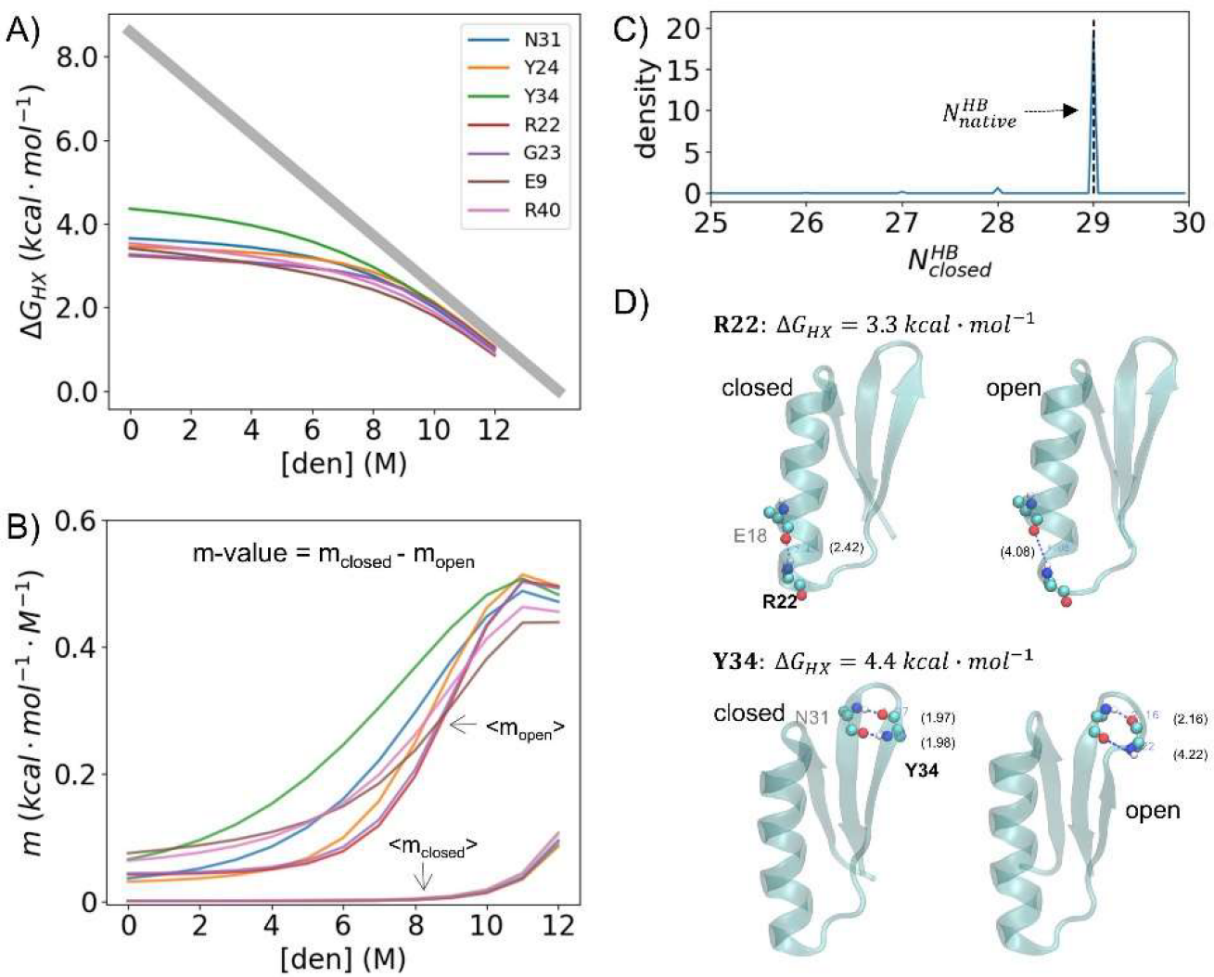
Local opening events of EHEE_rd2_0005 at *T* = 293 K. (A) HDX for seven residues that exchange via local or near-local openings. (B) Their *m*-values are decomposed into values for the closed and open states (*m*_closed_ and *m*_open_). The *m*_closed_ value of all of the local residues is close to 0 at low [urea] as all of the native H-bonds are formed >96% of the time when the H-bond of interest is formed. When the H-bond is broken, 79% and 16% of the time, 0 or 1 H-bond, respectively, is also broken. (C) Distribution of the number of H-bonds at *T* = 293 K. (D) Example structures for the local openings.

We investigated this possibility by examining *N*_closed_^HB^ for a variety of H-bonds having small *m*-values. In the *Upside* trajectories, we observe a narrow native ensemble with nearly all H-bonds being formed in the native well at low denaturant (*N*_closed_^HB^ ≈ *N*_total_^HB^) (Figures 12, S26, and S27). For example, the H-bond involving Arg22 of EHEE_rd2_0005 has an *m*-value that is 5% of the global *m*-value, and when the H-bond is formed, all other native H-bonds are formed >96% of the time. Additionally, when the H-bond of Arg22 is broken in the NSE, nearly all other H-bonds remain (i.e., 79% and 16% of the NSE have 0 or 1 additional H-bonds broken, respectively). For this and other sites having small *m*-values, the local opening events reflect the breaking of a single or a few H-bonds rather than a population redistribution within a broad native ensemble. Our simulations generally find that local openings occur at the termini of helices and strands or at turns. In helices, the donor and acceptor residues separate, whereas the NH becomes exposed in strands via individual crankshaft motions (Figure 12D).

### Testing for EX2 Behavior

An assumption in the calculation of stability from HDX data is that exchange is occurring in the thermodynamic EX2 limit where *k*_close_ ≫ *k*_chem_ and the rate is proportional to the fraction of time the NH is exchange competent, that is:
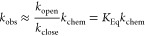
In the other EX1 limit where *k*_close_ ≪ *k*_chem_, exchange occurs every time the NH becomes exchange competent so that the observed rate matches the opening rate, *k*_obs_ = *k*_open_.

For EHEE_rd2_0005, we measured the refolding kinetics as a function of urea, tracking tryptophan fluorescence at 298 K, pH 7.54 to match the HDX condition. The folding rate extrapolated to 0 M urea was ∼1700 s^–1^, whereas *k*_chem_ for the measured NHs was between 9 and 26 s^–1^ for the five most stable sites. At the highest urea concentration (6 M), the corrected *k*_chem_ is 4–12 s^–1^ for these residues, while *k*_f_ = 390 s^–1^ (in 4 M urea, 1.25 M guanidine hydrochloride). Under the experimental conditions for HEEH_rd4_0097 (278 K, pD_read_ 4.6), *k*_chem_ is ∼0.008 s^–1^, which makes it highly likely that this small protein exchanges in the EX2 limit.^36^ Likewise, for Ub and the L50E variant, folding time constants are in sub-20 ms range (i.e., >50 s^−1^),^32,37^ whereas ⟨*k*_chem_⟩ = 2.4 s^–1^ at 273 K, pD_read_ 7.6 for the seven most stable sites. Hence, data for the proteins likely all are in the EX2 limit.

## Discussion

The goals of our study are to advance the *Upside* model’s ability to calculate the free energy surface and develop protocols for validating this and similar calculations using HDX and its denaturant dependence. A comparison involving the denaturant dependence provides a sensitive test of the veracity of the simulated ensemble as both the free energy and the structural content of the partially folded states must be correctly predicted. This task is particularly challenging for the larger openings as it requires that the simulations have low levels of residual H-bonded structure in the disordered regions. To achieve such cooperativity requires the stabilizing interactions to be strong enough to fold the protein while not overstabilizing residual structure.

We achieved moderate success with two small, designed proteins as well as Ub and an L50E variant, observing openings having probabilities as low as 1 part in a million for the two most stable proteins, EHEE_rd2_0005 and Ub. In the absence of denaturant, however, these rare events do not quite represent global unfolding. Because of an overprediction of the partially folded states, the Δ*G*_HX_ values for the most stable NHs in the simulations are below the experimentally determined values for the sites that likely exchange by global unfolding by 0.2–1.5 kcal mol^–1^. With urea, these states are destabilized, and the HDX shifts from being dominated by local and subglobal openings to occurring via larger-scale openings, including global exchange, in agreement with experiment.

*Upside*’s performance is the product of our ConDiv training procedure that was trained for stable native and disordered unfolded states. The dual-target training increased folding cooperativity and reduced the amount of residual H-bonded structure, which is essential for accurately predicting HDX patterns. Other improvements included energy terms related to backbone and side chain desolvation.

We are able to reversibly fold EHEE_rd2_0005 and HEEH_rd4_0097 but did not achieve this level of success for Ub and the L50E variant. For these larger proteins, we adopted a strategy of calculating HDX using only the portion of the trajectories that connects the NSE to the DSE, mimicking actual HDX measurements. The general agreement, especially for the L50E variant, argues that this approach does provide a way forward for handling larger proteins, especially for describing the lower energy, smaller-scale opening events.

Other challenges exist in predicting HDX data. There must be sufficient sampling that the ensemble properly reflects the Boltzmann weighting, especially for the rare opening events. The use of enhanced sampling methods including replica exchange combined with reweighting methods such as MBAR^22^ is extremely useful.

Another challenge relates to the observation of rare events. Their observation in an HDX experiment requires that no lower energy opening exists as it would dominate the exchange process. Hence, to match the experimental observation of a high energy species requires the model both accurately simulate the rare species and, at the same time, not have any lower energy states where those NHs are not involved in H-bonding. Hence, reproducing HDX data requires an accurate calculation of the free energy surface at both high and low energies.

This issue becomes more problematic with more stable proteins as overprediction of exchange competant states becomes more likely as the energy gap between the DSE and NSE increases. This effect can be seen in our poorer prediction of HDX for wild-type ubiquitin as compared to the L50E variant, which is 5 kcal·mol^–1^ less stable.

From the experimental perspective, the conversion of experimental HDX rates to Δ*G*_HX_ requires knowledge of the intrinsic rate *k*_chem_. The standard intrinsic rates were obtained using peptides in 0.5 M KCl, which is appropriate for unfolded chains.^38^ While deviations in *k*_chem_ have been observed in unusual electrostatic environments^39,40^ or with highly charged proteins,^40^ we believe these effects are likely to have minimal impact on the large-scale unfolding events we are investigating as the charge density is lower in these expanded states. Nevertheless, these effects could affect the comparison between local unfolding events. Future HDX studies may be conducted in the presence of divalent cations to reduce these effects.^41^

### Previous HDX Prediction Studies

The challenge of calculating a conformational ensemble that determines the free energy surface and HDX pattern is quite different from the task of predicting Δ*G*_HX_. A variety of strategies have predicted Δ*G*_HX_ using properties of the native state^23^ sometimes augmented by HDX enhancing motions.^42,43^ HDX data have also been used to improve computational ensembles.^44−46^ For the purposes of our study, however, we are focused on *de novo* HDX calculations that are based solely on the NH protection levels in a predicted Boltzmann ensemble.

Generally, at least one amide proton exchanges with the stability and denaturant dependence consistent with total unfolding.^4,47^ This observation implies that HDX for such sites occurs from a highly expanded state. Hence, models where exchange is predicted to occur from near-native or collapsed, H-bonded conformations do not accurately describe the Boltzmann ensemble, regardless of how well they may predict Δ*G*_HX_ in the absence of denaturant.

All-atom simulations are well suited to predict HDX.^9−14^ Although improvements have been made,^7,8^ these simulations generally have too much residual structure to be consistent with the strong denaturant dependence seen in HDX data. Other approaches with the potential to predict Boltzmann ensembles and HDX patterns include coarse-grained Go models^48^ and COREX.^5,49^ The latter method generates energy-weighted ensembles where each site is considered binarily as either native or unfolded in windows of 6+ residues.^5,49^

In their comparison of COREX to HDX data,^6^ Hilser et al. suggested that the NSE is broad with 10–20% of the buried surface exposed. Within the context of this broad ensemble, they proposed that small *m*-values arise when the average exposure in the subensemble having the specific H-bond closed matches the exposure in the subensemble where the H-bond is open: that is, *m* = *m*_closed_ – *m*_open_ ≈ 0.^5^ As discussed earlier, such a broad NSE is not observed in the *Upside* simulations. Rather, we find that small *m*-values reflect H-bonds predominantly breaking as singletons due to local deformations at the ends of secondary structures and turns (Figures 12). This observation supports the standard view that local unfolding events occur with the breaking of only one or a few H-bonds^3^ and the native well is a relatively narrow ensemble (Figures S26 and S27).

## Conclusion

We found that the prediction of the free energy surface and validation using HDX is extremely challenging and hence provides a very rigorous test of the accuracy of an energy function and sampling engine. The pair must predict the probability and structural content of rare events while having a DSE with minimal residual structure and avoid overpredicting intermediate-level fluctuations. In this light, we view the outcome of the present study to be commendable, although we appreciate that there is room for improvement.

We improved our performance by training the energy function to simultaneously fold proteins and have an unstructured DSE. This results in more realistic denatured states and increased folding cooperativity, which improves the match to HDX data. The dual-target training procedure also improved our ability to predict protein stability even though this was not an explicit goal, suggesting that the training procedure should be useful in improving other energy functions.

## Methods

MD simulation and sampling can be found in the Supporting Information Simulation Details and Sampling. Protein sequences, expression, HDX/NMR, and kinetic studies can be found in the Supporting Information Experimental Methods. The *Upside* package is available for download at https://github.com/sosnicklab/upside-md.

## Supporting Information Available

Detailed discussion about the energy function improving, dual-objective contrastive divergence training, temperature calibration, stability prediction, simulation/sampling methods, and experimental methods (PDF)
