# Supplementary material for: Prediction and Validation of a Protein’s Free Energy Surface Using Hydrogen Exchange and (Importantly) Its Denaturant Dependence
Source: J Chem Theory Comput. 2021 Dec 22;18(1):550–61. doi: 10.1021/acs.jctc.1c00960 (PMC8757463; doi:10.1021/acs.jctc.1c00960)
Supplement: Supplementary file 1 — ct1c00960_si_001.pdf [file ct1c00960_si_001.pdf]

Supporting information for

Prediction and validation of a protein's free energy surface using hydrogen exchange and (importantly) its denaturant dependence

Xiangda Peng, Michael Baxa, Nabil Faruk, Joseph R. Sachleben, Sebastian Pintscher, Isabelle A Gagnon, Scott Houliston, Cheryl Arrowsmith, Karl F. Freed, Gabriel J. Rocklin, Tobin R. Sosnick

Improving the energy function

Ramachandran Energy. In the original *Upside* energy function [1,2], our Ramachandran energy term combined the TCB (turn, coil, and bridge states, as defined by DSSP[3]) Ramachandran frequencies and the sheet frequencies to balance the helix/sheet tendency:

$$RAMA_{aa_i}(\varphi_i, \phi_i) = \frac{(1-g)RAMA^{TCB}_{aa_{i-1},aa_i,aa_{i+1}}(\varphi_i,\phi_i) + g\times e^{-\gamma} RAMA^{sheet}_{aa_{i-1},aa_i,aa_{i+1}}(\varphi_i,\phi_i)}{(1-g)+g\times e^{-\gamma}} \qquad \text{(eq. S1)}$$

The parameter γ controls the proportion of β-sheet angles in this model. However, the helix/sheet tendency of different amino acid is not exactly the same, so γ is amino acid dependent:

$$RAMA_{aa_i}(\varphi_i, \phi_i) = \frac{(1-g)RAMA^{TCB}_{aa_{i-1},aa_i,aa_{i+1}}(\varphi_i,\phi_i) + g\times e^{-\gamma_{aa_i}} RAMA^{sheet}_{aa_{i-1},aa_i,aa_{i+1}}(\varphi_i,\phi_i)}{(1-g)+g\times e^{-\gamma_{aa_i}}} \qquad \text{(eq. S2)}$$

The H-bond term. Our H-bond potential is a composite of a strength s_{hbond} and H-bond score for the proton and the oxygen:

$$V_{hbond,i} = s_{hbond} \times \left(hbscore_i^H + hbscore_i^O\right) \qquad \text{(eq. S3)}$$

where

$$hbscore_i^H = 1.0 - \prod_{j\neq i}\left(1.0 - h_{ij}\right) \qquad \text{(eq. S4)}$$

$$h_{ij} = f_{redial}(r_{HO}) f_{angular}(a_{HOC}) f_{angular}(a_{NHO}) \qquad \text{(eq. S5)}$$

where $f_{redial}(r)$ and $f_{angular}(a)$ are:

$$f_{redial}(r) = sigmoid\left(\frac{r-1.4}{0.10}\right) sigmoid\left(\frac{2.5-r}{0.125}\right) \qquad \text{(eq. S6)}$$

$$f_{angular}(a) = sigmoid\left(\frac{-0.682-cos(a)}{0.05}\right) \qquad \text{(eq. S7)}$$

The $hbscore_i^O$ is similarly defined.

Due to geometric constraints or changes in pKa of the NH, the strength of H-bonds in different secondary structures may not be the same. For example, H-bonds are more linear in antiparallel strands than in parallel strands or helices. Accordingly, the strength of H-bond is made dependent on Ramachandran angle:

$$s_{hbond,i} = f^{helix}(\varphi_i, \phi_i)s_{hbond}^{helix} + f^{sheet}(\varphi_i, \phi_i)s_{hbond}^{sheet} + f^{turn}(\varphi_i, \phi_i)s_{hbond}^{turn} \quad \text{(eq. S8)}$$

The function $f^{helix/sheet/turn}$ ranges from 0 to 1, and is used to determine the current secondary structure according to the Ramachandran angles, as shown in **Fig. S1**. ConDiv training return an H-bond energy for helix, strand and turn of s_{hbond}= -1.96, -1.95, and -1.77 k_BT, respectively.

To mimic the effects of polarizability in a peptide group which occurs upon H-bond formation, the energy of a second H-bond is increased by a factor of $s_{2ndhbond}$:

$$ds_{hbond,i}^{H} = ds_{hbond,i-1}^{O} = s_{2ndhbond} \times \left(hbscore_i^H + hbscore_{i-1}^O - 1\right) \quad \text{(eq. S9)}$$

The $s_{2ndhbond}$ value is -0.41 k_BT, obtained using ConDiv training.

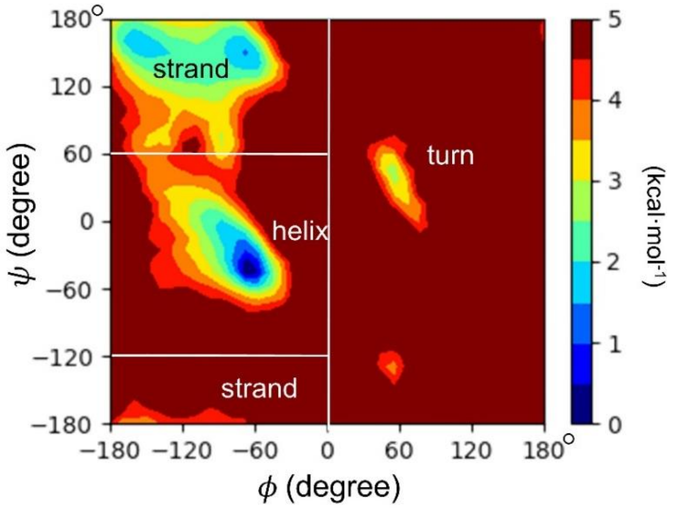

Fig S1. H-bond energy depends on secondary structure type, as defined here.

Desolvation terms. In our original 2018 study, *Upside*'s multibody desolvation term is based on the burial level, the number of side chain beads in a hemisphere centered at the C_β atom (H_α atom for GLY) with an orientation along the C_α-C_β direction, and the radius is 9 Å, defined by sigmoid border which decreases from 1 to 0 when the radius increases from 8 to 10 Å. Now, the beads are weighted based on the amino acid type to describe the physio-chemical properties of amino acids when counting the burial level. These weight factors are trained by ConDiv, showing the correlation with hydrophobicity, total mass, and a linear combination of the two (**Fig. S2**).

The ConDiv training procedure establishes the mapping between the burial level and the desolvation energy. In the 2018 version of *Upside*, the mapping is based on the spline-like function which is replaced by the sigmoid-like function to increase the smoothness in this work.

The solvation of the backbone and the H-bonds stabilizes the DSE. This is important for minimizing the amount of residual structure with is essential for accurate prediction of HDX and

folding cooperativity. A multibody term is added which is similar to the term for side chain, but with the C_α-C_β hemisphere moved to the N-H and C-O bond vectors to determine the solvation of the backbone NH or CO groups.

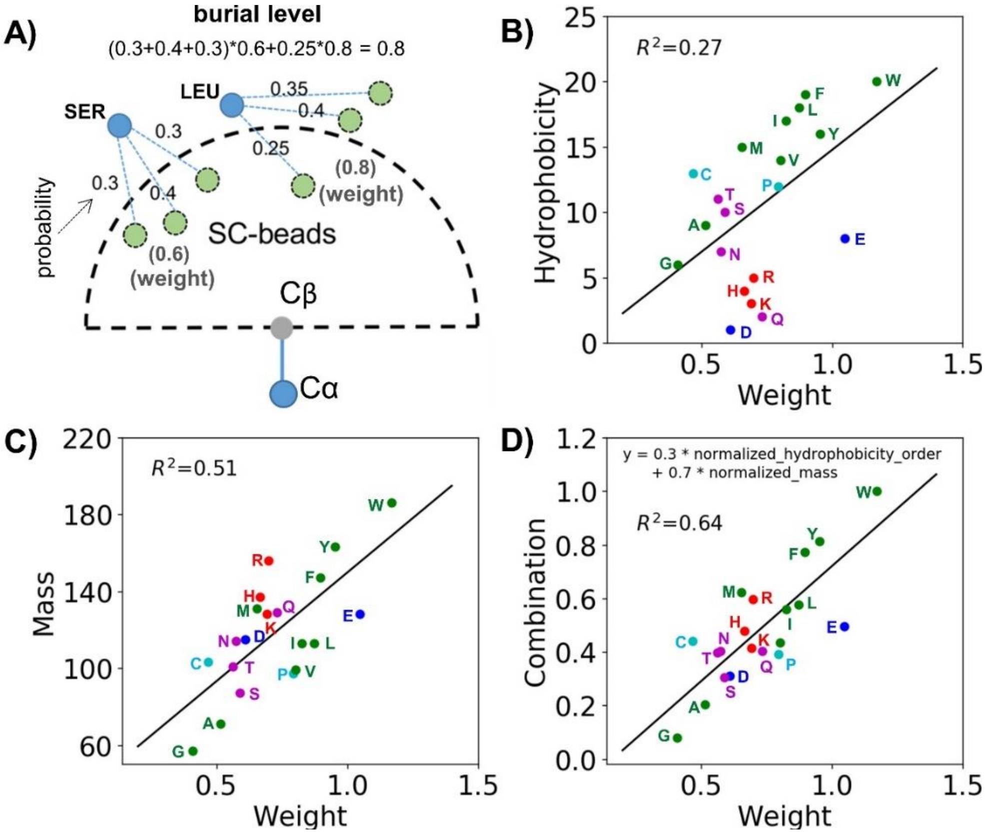

Fig S2. Side chain buried level. A) Different amino acid types are given different weights to capture the chemical contribution to desolvation, here illustrated with a SER (0.6 weight) and LEU (0.8 weight). Each residue is represented by a side chain bead that can be in three positions with a total probability summing to unity. For LEU, the two positions having probabilities of 0.35 and 0.4 are too far from the Cβ to contribute. The weights are learned in ConDiv training and correlate with **B**) side chain hydrophobicity, **C**) total mass, and **D)** a linear combination.

Parameterization by Contrastive Divergence

Using ConDiv method, all parameters can be optimized simultaneously to stabilize NSE[2]. In each training step, there are two ensembles from two different types of simulations: one restrained to the native structure (native ensemble) and another that is allowed to diffuse during simulations (free ensemble). For an imperfect force field, the unrestrained ensemble will diffuse away from the native state. By comparing the difference of the derivative of the potential energy with respect to the force field parameters in the two ensembles, the parameters can be iteratively updated to preferentially stabilize the native ensemble (**Fig. 2**).

$$\alpha_{i+1} - \alpha_i = \varepsilon\left(-\left\langle\frac{dV}{d\alpha}(native)\right\rangle + \left\langle\frac{dV}{d\alpha}(x_i)\right\rangle\right) \qquad \text{(eq. S10)}$$

For the new **FF2**, we added a second objective related to training the DSE to be a self-avoiding random walk (SARW). The SARW distribution is generated by running *Upside* without H-bonds bonding, side chain and solvation terms. A replica close to the T_m is used to select the training ensemble (refer as $x_{unfolded}$) containing the frames whose Rg is greater than $2/3\,Rg_{native} + 1/3\,Rg_{SARW}$ (**Fig. S3**). Then, the equation used for parameter update is changed to:

$$d\alpha = d\alpha_{NSE} + \lambda \times d\alpha_{DSE} \qquad \text{(eq. S11)}$$

$$d\alpha_{NSE} = \varepsilon\left(-\left\langle \frac{dV}{d\alpha}(native)\right\rangle + \left\langle \frac{dV}{d\alpha}\left(x_{folded}\right)\right\rangle\right) \qquad \text{(eq. S12)}$$

$$d\alpha_{DSE} = \varepsilon\left(-\left\langle \frac{dV}{d\alpha}(SARW)\right\rangle + \left\langle \frac{dV}{d\alpha}\left(x_{unfolded}\right)\right\rangle\right) \qquad \text{(eq. S13)}$$

The value of λ in is empirically set to 0.3. The second training objective can be viewed as a regularizer that simultaneously makes the residual interactions as weak as possible while stabilizing the NSE. We expect that this strategy can reduce the risk of overfitting and get a better balance between entropy and potential energy.

The dual-target ConDiv training procedure was run for 456 proteins to train new potential energy parameters. These proteins were divided into 19 batches, and a total of 76 ConDiv iterations (4 cycles) were executed. For each protein, we perform one restrain simulation (to native state), one SARW simulation and 12 replica simulations (temperature from 0.8 to 1.1), the replica with lowest temperature is selected for training NSE, and the unfolded conformation in the replica close to Tm is selected for training DSE (**Fig. S3**). The length of these simulations is 8000 *Upside* time per training step. The second half of the trajectory is used to calculate the average derivative of the force field parameters with respect to the potential energy. The initial learning rate of the "fine-tuning" stage in Ref [2] is selected to train **FF2**. Other details in the training have been Ref [2]. Other details in training for **FF1** [2] were followed by this work.

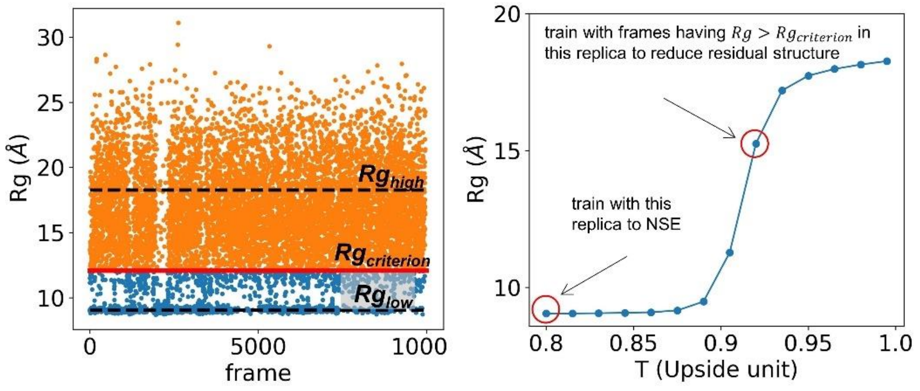

Fig S3. Selection of a DSE and NSE for dual objective training. For a trajectory, the red line defines the minimum $Rg_{criterion} = {}^{2}/_{3}\,Rg_{low} + {}^{1}/_{3}\,Rg_{high}$, which is used for selecting the frames for the ensemble used to train the DSE (the orange points). A low temperature replica is used for NSE training.

Folding Accuracy

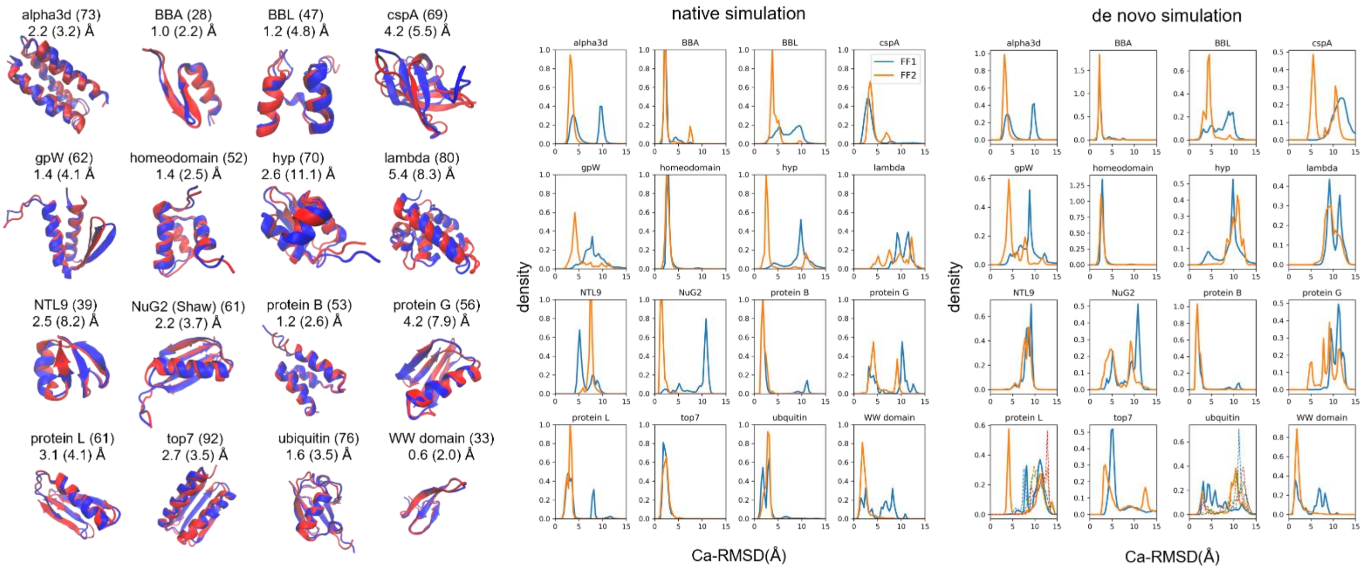

Fig S4. Predicted structures and Cα-RMSD distributions for 16 small proteins. The structure (red) with the lowest Cα-RMSD is selected for comparison with native structure (blue). The lowest Cα-RMSD is provided along with the value for the centroid of largest cluster (in parentheses, the DBSCAN[4,5] method is used to do the clustering based on the contact map between Cα with a 10 Å cutoff). Cα-RMSD distribution of the validation set starting from either a native (middle) or unfolded conformation (right) for FF1[2] and FF2. For some proteins, RMSD calculations exclude some residues at the amino- and/or carboxy-termini to account for possible disorder (BBA:1-3,28; BBL:1-6; cspA:1-3; gpW:1,56-62; homeodemain:1-4,50-52; hyp:1-5; lambda:1,2; NuG2:1-4; protein B:1-6,52,53; ubiquitin:1,74-76; WW domain:1-4,29-31).

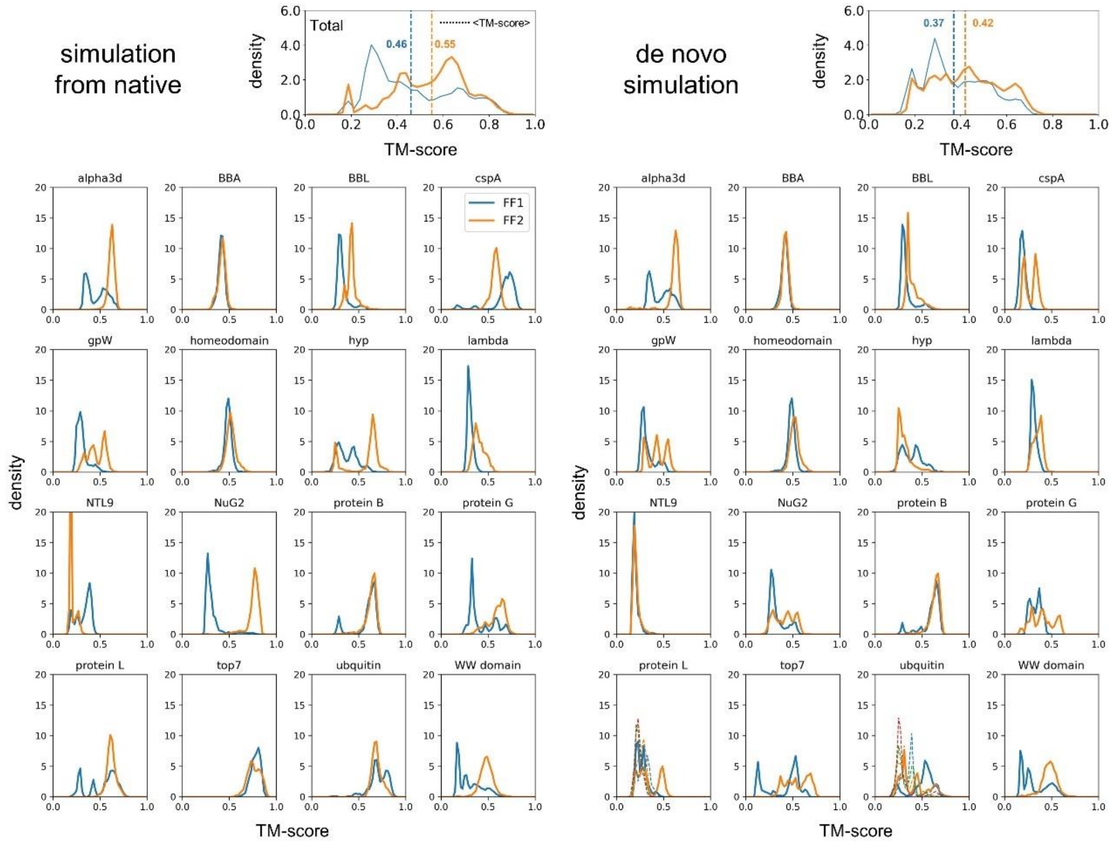

Fig S5. TM-score [6] distributions of the validation set starting from either a native (middle) or unfolded conformation (right) for FF1[2] and FF2. The native state is selected as the reference structure. The average distribution of 16 small proteins is displayed on the top.

Cooperativity

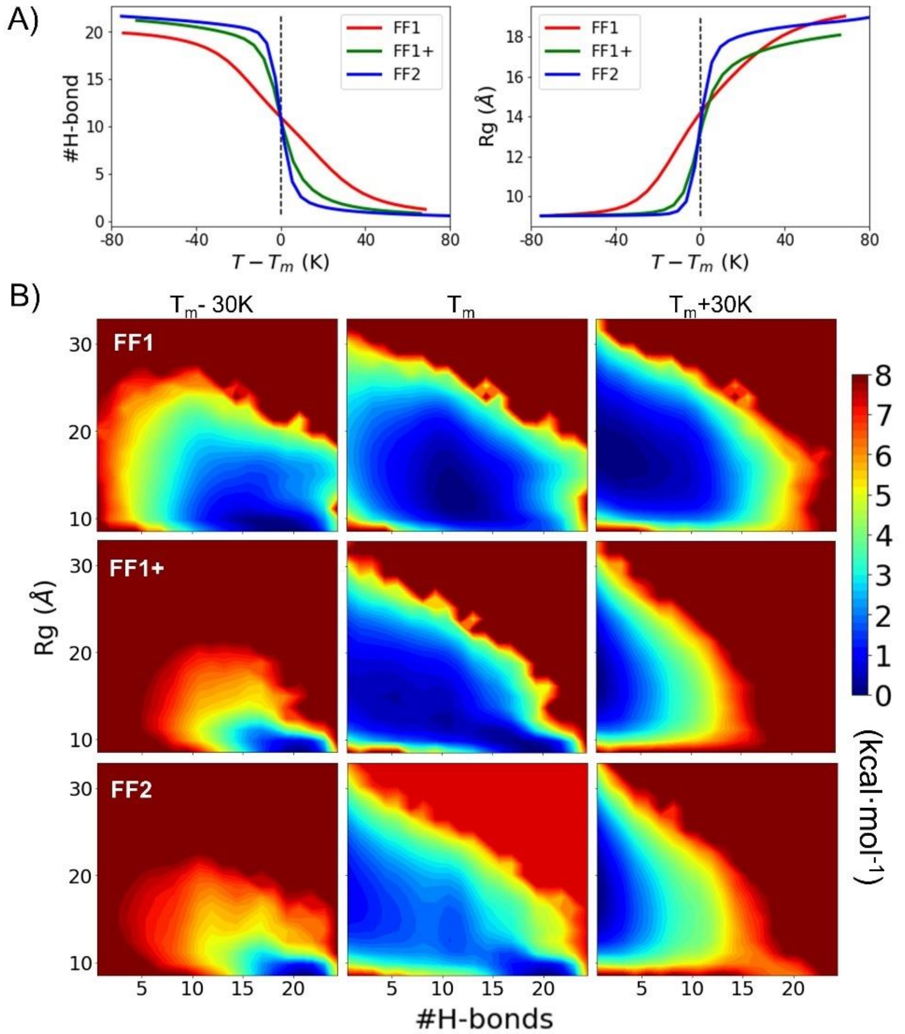

Fig S6. Comparison of Upside force fields. A) Melting curves highlighting the increased cooperativity of **FF1+** and **FF2**. **B)** Free energy surfaces at T below, at, and above the T_m. **FF1** has unrealistically high levels of of backbone H-bonds in the DSE whereas **FF2** has low levels along with a well-defined native state. Energy scale is proportional to -RT ln(Population) (low energy implies higher population).

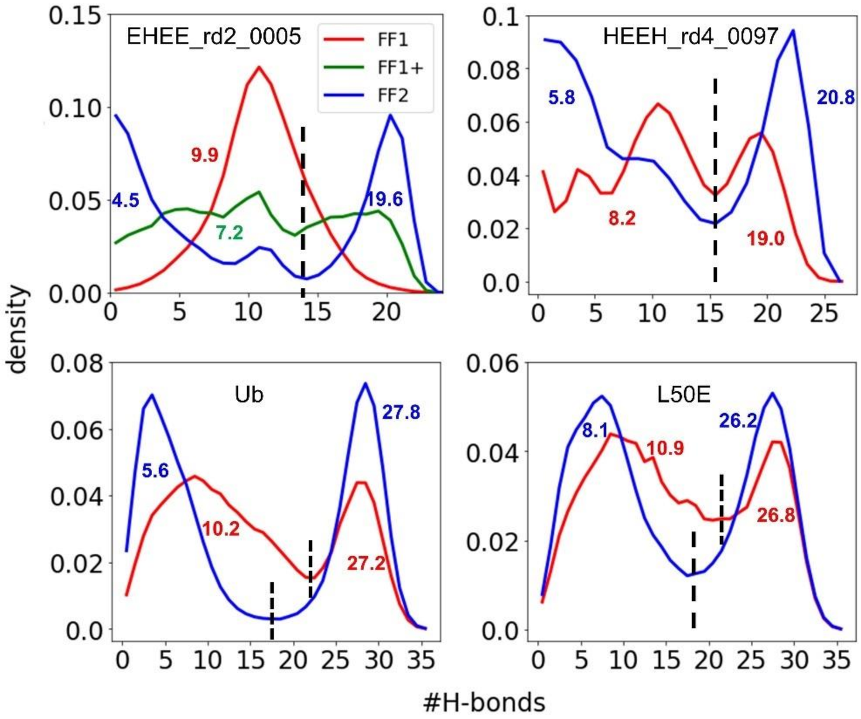

Fig S7. H-bond distributions at T_m for different *Upside* force fields. Each sub-plot lists the average number of H-bonds in the DSE and NSE. The separation between the NSE and DSE is denoted with a vertical black dashed line located at the minimum value between the two peaks, except for **FF1** with EHEE_rd2_0005 where the distribution is not bimodal.

Temperature calibration and Stability prediction

In order to calibrate the temperature and energy unit of *Upside*, we define a conversion factor *c* that can convert the energy unit of *Upside* to kcal·mol^{-1},

$$c \times R_{Upside} T_{Upside} \;=\; 0.593\; kcal \cdot mol^{-1} \quad (\text{when } T = 298\; K) \qquad \text{(eq. S14)}$$

Based on free energy equation

$$\Delta G_{kcal\cdot mol} = c \times \Delta G_{Upside} = c \times \left(\Delta H_{Upside} - T\Delta S_{Upside}\right) = c \times \Delta H_{Upside} - \frac{0.593}{R_{Upside}} \Delta S_{Upside}$$

(eq. S15)

the conversion factor *c* can be determined by

$$c = \frac{\frac{0.593}{R_{Upside}} \Delta S_{Upside} + \Delta G_{kcal\cdot mol}}{\Delta H_{Upside}} \qquad \text{(eq. S16)}$$

where $R_{Upside} = 1.0$, the $\Delta G_{kcal\cdot mol}$ comes from the average stability of 13 Rosetta-designed mini-proteins at 298 K[7]. For each protein, temperature replica exchange molecular dynamics (T-REMD)

simulations were run to generate melting curves that were fit assuming a two-state U-to-N model (**Fig. S8**). And the ΔH_{Upside} ΔS_{Upside} are obtained from the fitting. (**Table S1**)

Table S1. The mini proteins for temperature and unit calibration

	ΔG_{exp} (kcal·mol^{-1})	FF1 (*Upside* unit)				FF1+ (*Upside* unit)				FF2 (*Upside* unit)			
		ΔH_{Rg}	ΔS_{Rg}	ΔH_{hb}	ΔS_{hb}	ΔH_{Rg}	ΔS_{Rg}	ΔH_{hb}	ΔS_{hb}	ΔH_{Rg}	ΔS_{Rg}	ΔH_{hb}	ΔS_{hb}
EEHEE_rd3_1049	3.18	62.2	61.1	47.3	46.6	106.8	96.6	91.0	82.7	82.0	90.7	70.9	78.6
EHEE_rd1_0284	4.70	45.0	45.1	39.1	39.2	63.9	60.8	63.1	60.2	126.8	137.6	118.6	128.9
EHEE_rd1_0407	3.50	15.9	16.3	20.5	20.2	68.6	63.3	66.0	61.1	111.3	122.6	112.8	124.5
EHEE_rd2_0303	1.94	92.7	90.9	87.5	85.9	76.2	71.0	87.4	81.7	46.0	50.7	46.3	51.2
HEEH_rd2_0779	1.22	41.3	40.4	22.6	22.4	28.9	28.2	33.6	32.9	41.2	47.1	43.5	49.9
HEEH_rd4_0094	1.78	39.2	38.7	37.8	37.7	52.7	48.4	49.6	46.3	44.2	50.4	49.7	57.0
HEEH_rd4_0097	2.65	52.1	49.0	46.9	44.3	49.2	45.6	50.5	47.3	70.3	78.0	70.5	78.4
HEEH_rd4_0349	1.84	43.7	41.9	42.0	40.4	40.6	38.0	45.0	42.6	44.7	50.9	51.4	58.8
HHH_rd1_0092	1.02	53.2	52.1	47.5	46.5	56.6	52.0	54.1	49.8	67.6	75.3	61.9	69.1
HHH_rd1_0142	2.81	73.0	69.6	65.3	62.4	84.8	79.4	78.3	73.4	57.2	64.4	55.8	62.7
HHH_rd2_0134	4.53	51.0	48.4	41.4	39.2	69.5	64.9	64.0	59.8	88.1	98.7	81.1	90.8
HHH_rd3_0006	1.70	50.7	47.4	47.1	43.8	67.5	61.6	55.9	51.0	64.3	72.1	53.9	60.0
HHH_rd3_0008	4.34	41.0	39.4	35.2	33.9	54.6	51.0	56.1	52.7	66.3	76.2	64.1	73.6
average	2.71	50.9	49.2	44.6	43.3	63.0	58.5	61.1	57.0	70.0	78.1	67.7	75.7
c		0.627		0.636		0.593		0.598		0.700		0.702	

Based on the eq. S14 and the determined *c* value, the *Upside* temperature unit of 1.0, 0.94 and 0.85 is equivalent to 298K for **FF1**, **FF1+** and **FF2** respectively (**Fig. S9**).

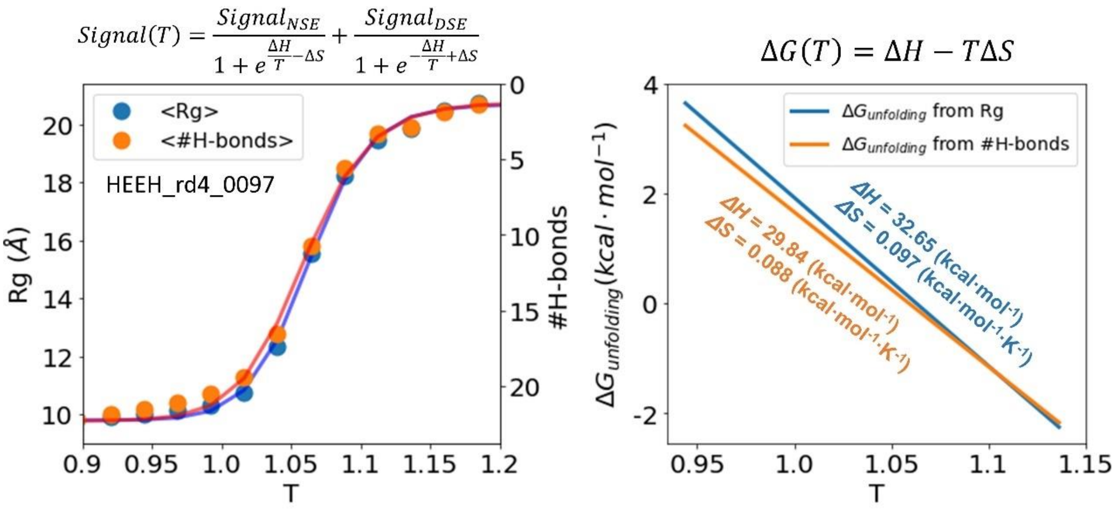

Fig S8. The free energy is obtained from the simulated melting curve of either the fraction of H-bonds or the Rg using $\Delta G(T) = \Delta H - T\Delta S$. The predicted melting curve and ΔG(T) for HEEH_rd4_0097 is shown as an example.

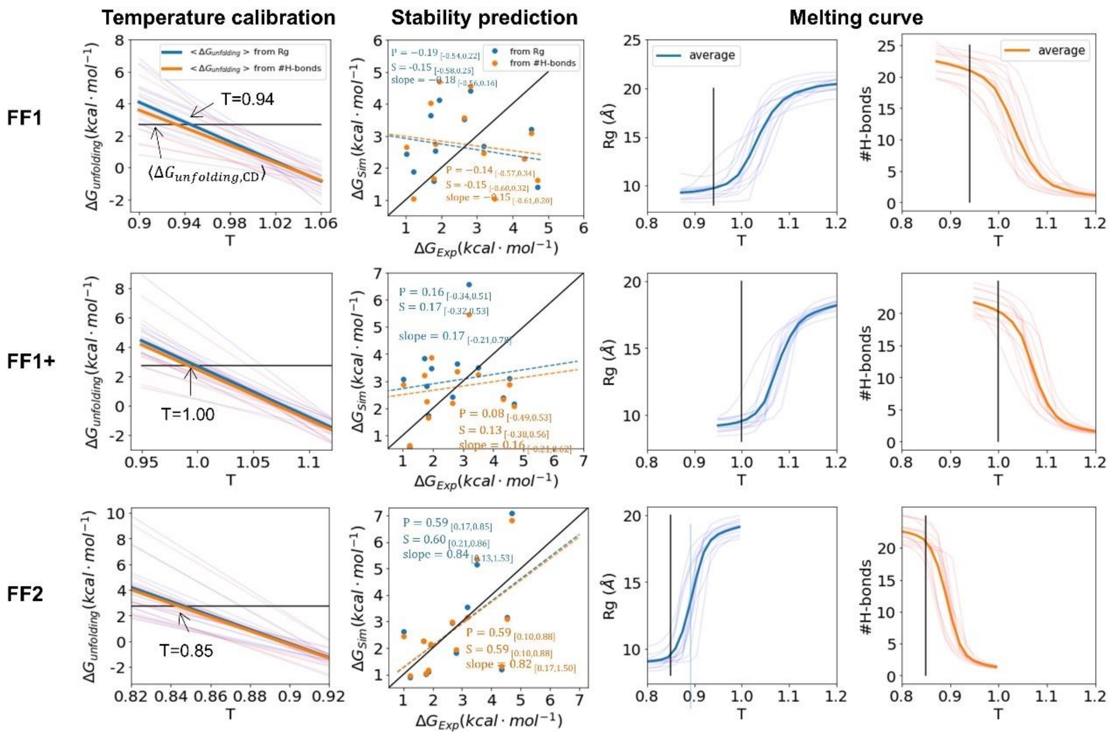

Fig S9. Calibrating *Upside* temperature scale using experimental data. The simulated melting curves tracking the number of H-bonds are fit to determine ΔG(T) (**Fig. S8)** for a set of 13 small Rosetta designed proteins (**Table S2**) with known stabilities at 298 K[8] (dashed lines are for individual proteins, solid lines are for the average). The single *Upside* temperature that produces the best correlation with the experimental stabilities is assigned a value of 298 K (T=0.86 for **FF2**). The results for melting curves for the R_g produce very similar results. In the stability prediction plots, the best fit line, slope, and Pearson (P) and Spearman (S) correlation coefficient are given along with the 90% confidence interval. the confidence interval is estimated by bootstrapping method.

Simulation details and sampling

Benchmarking the simulations.

To examine the performance of **FF2**, we used a test set consisting of 16 small proteins [2] (**Table S2)**. For each protein, a simulation was run starting from native state (native simulation) and from the unfolded state (de novo simulation). The replica exchange algorithm with 14 replicas was used to enhance sampling. The temperature and simulation length are listed in **Table S2**.

Table S2. The simulation time and temperature (*Upside* unit) for the proteins in folding test set.

Name	Type	Simulation time	Temperature
alpha3d	native	2713400	0.900,0.908,0.916,0.924,0.932,0.940,0.947,0.955,0.963,0.970,0.978,0.985,0.993,1.000

	denovo	3041800	0.900,0.908,0.916,0.924,0.932,0.940,0.947,0.955,0.963,0.970,0.978,0.985,0.993,1.000
BBA	native	2893100	0.650,0.695,0.737,0.777,0.815,0.852,0.887,0.920,0.952,0.984,1.014,1.043,1.072,1.100
	denovo	4000000	0.650,0.695,0.737,0.777,0.815,0.852,0.887,0.920,0.952,0.984,1.014,1.043,1.072,1.100
BBL	native	2083400	0.700,0.733,0.764,0.795,0.824,0.852,0.879,0.905,0.931,0.956,0.980,1.004,1.027,1.050
	denovo	8000000	0.700,0.733,0.764,0.795,0.824,0.852,0.879,0.905,0.931,0.956,0.980,1.004,1.027,1.050
cspA	native	2968600	0.750,0.768,0.786,0.803,0.820,0.837,0.853,0.869,0.885,0.901,0.916,0.931,0.946,0.960
	denovo	6369200	0.750,0.768,0.786,0.803,0.820,0.837,0.853,0.869,0.885,0.901,0.916,0.931,0.946,0.960
gpW	native	2845900	0.800,0.813,0.825,0.837,0.849,0.861,0.872,0.884,0.895,0.906,0.918,0.929,0.939,0.950
	denovo	5919500	0.800,0.813,0.825,0.837,0.849,0.861,0.872,0.884,0.895,0.906,0.918,0.929,0.939,0.950
homeodomain	native	3115600	0.870,0.881,0.891,0.902,0.912,0.922,0.932,0.942,0.952,0.962,0.972,0.981,0.991,1.000
	denovo	3078800	0.870,0.881,0.891,0.902,0.912,0.922,0.932,0.942,0.952,0.962,0.972,0.981,0.991,1.000
hyp	native	2634000	0.800,0.817,0.834,0.850,0.866,0.882,0.898,0.913,0.928,0.943,0.956,0.972,0.986,1.000
	denovo	5522600	0.800,0.817,0.834,0.850,0.866,0.882,0.898,0.913,0.928,0.943,0.956,0.972,0.986,1.000
lambda	native	2533700	0.780,0.797,0.814,0.830,0.847,0.862,0.878,0.893,0.908,0.923,0.938,0.952,0.966,0.980
	denovo	2573700	0.780,0.797,0.814,0.830,0.847,0.862,0.878,0.893,0.908,0.923,0.938,0.952,0.966,0.980
NTL9	native	4000000	0.680,0.710,0.738,0.766,0.792,0.818,0.843,0.867,0.891,0.914,0.936,0.958,0.979,1.000
	denovo	8000000	0.680,0.710,0.738,0.766,0.792,0.818,0.843,0.867,0.891,0.914,0.936,0.958,0.979,1.000
NuG2	native	3143500	0.820,0.832,0.843,0.854,0.865,0.876,0.887,0.898,0.909,0.919,0.930,0.940,0.950,0.960
	denovo	6612200	0.820,0.832,0.843,0.854,0.865,0.876,0.887,0.898,0.909,0.919,0.930,0.940,0.950,0.960
protein B	native	3894500	0.800,0.813,0.825,0.837,0.849,0.861,0.872,0.884,0.895,0.906,0.918,0.929,0.939,0.950
	denovo	4000000	0.800,0.813,0.825,0.837,0.849,0.861,0.872,0.884,0.895,0.906,0.918,0.929,0.939,0.950
protein G	native	3368700	0.800,0.812,0.823,0.834,0.846,0.857,0.867,0.878,0.889,0.899,0.910,0.920,0.930,0.940
	denovo	7804600	0.800,0.812,0.823,0.834,0.846,0.857,0.867,0.878,0.889,0.899,0.910,0.920,0.930,0.940
protein L	native	3553200	0.800,0.812,0.823,0.834,0.846,0.857,0.867,0.878,0.889,0.899,0.910,0.920,0.930,0.940
	denovo*	7763800	0.800,0.812,0.823,0.834,0.846,0.857,0.867,0.878,0.889,0.899,0.910,0.920,0.930,0.940
top7	native	1792821	0.950,0.961,0.971,0.982,0.992,1.002,1.012,1.022,1.032,1.042,1.051,1.061,1.071,1.080
	denovo	2147700	0.910,0.921,0.931,0.942,0.952,0.962,0.972,0.982,0.992,1.002,1.011,1.021,1.031,1.040
ubquitin	native	2429200	0.800,0.812,0.823,0.834,0.846,0.857,0.867,0.878,0.889,0.899,0.910,0.920,0.930,0.940
	denovo*	8028200	0.800,0.812,0.823,0.834,0.846,0.857,0.867,0.878,0.889,0.899,0.910,0.920,0.930,0.940
WW domain	native	4000000	0.700,0.733,0.764,0.795,0.824,0.852,0.879,0.905,0.931,0.956,0.980,1.004,1.027,1.050
	denovo	4000000	0.700,0.733,0.764,0.795,0.824,0.852,0.879,0.905,0.931,0.956,0.980,1.004,1.027,1.050

* Five independent simulations were run. In **Fig. S4** (right), the simulation with the lowest RMSD is shown with the solid orange line. Others are indicated by dashed lines.

Mini proteins designed by Rosetta

We performed REMD simulations on EHEE_rd2_0005, HEEH_rd4_0097 and another 12 small proteins Rosetta designed (**Table S3**) using **FF1**, **FF1+** and **FF2**. The trajectories of EHEE_rd2_0005 and HEEH_rd4_0097 were used to calculate the FES and HDX and compare with experimental data. The simulations of HEEH_rd4_0097 and the other 12 small proteins are used to calculate the stability[7] for purposes of temperature calibration. We use the design targets[7] as the initial structure in the T-REMD simulations with 14 replicas per protein. The temperature range is selected to cover the denaturation profile. The temperature range is 0.848 to 1.208, 0.95 to 1.22, and 0.8 to 0.995 for the simulations with **FF1**, **FF1+** and **FF2** respectively. The simulation length ranges from 4 - 12 M of *Upside* time units to allow for multiple reversible folding/unfolding transitions (**Table S3**). For all analyses, the first 500 frames (50000 *Upside* time units) are discarded to reduce the impact of initial conditions.

Table S3. The simulation time (*Upside* unit) for mini proteins.

	FF1	FF1+	FF2
EHEE_rd2_0005	16000000	4000000	4000000
HEEH_rd4_0097	16000000	4000000	4000000
EEHEE_rd3_1049	4000000	4000000	8000000
EHEE_rd1_0284	4000000	4000000	11068000
EHEE_rd1_0407	4000000	3680000	7850000
EHEE_rd2_0303	8000000	4000000	8000000
HEEH_rd2_0779	4000000	4000000	4000000
HEEH_rd4_0094	4000000	4000000	4000000
HEEH_rd4_0349	4000000	4000000	4000000
HHH_rd1_0092	4000000	4000000	4000000
HHH_rd1_0142	4000000	4000000	4000000
HHH_rd2_0134	4000000	4000000	4000000
HHH_rd3_0006	4000000	4000000	4000000
HHH_rd3_0008	8000000	4000000	12000000

We also performed constant temperature dynamics simulations to check the reversibility of folding and unfolding for EHEE_rd2_0005 and HEEH_rd4_0097 near the melting temperature. We choose T=0.916 (321K) for EHEE_rd2_0005, and T=0.897 (314K) for HEEH_rd4_0097, with a simulation time of 8000000 of *Upside* time units.

Ub and L50E

For both Ub and L50E, three independent replica exchange simulations are performed using **FF2** with 14 replicas from 0.8 to 1.02 (280 to 358 K) and 1ubq.pdb as the initial structure. The length of the individual simulation is >2400000 *Upside* time units. These trajectories are used to obtain the free energy surfaces and calculate HDX after discarding the first half of the trajectories (22,000 frames) for Ub and 5000 frames for L50E.

For all the simulations, a Verlet integration is used with a step size of 0.009 *Upside* time units and random number generator to implement the Langevin dynamics using Ornstein-Uhlenbeck thermostat[8] with a thermalization time scale of 5.0 time units. Coordinates are output at a interval of 100 *Upside* time units. In all replica exchange simulations, the exchange attempts are executed every 10 *Upside* time units.

Reweighting

Since HDX is sensitive to all species in the Boltzmann ensemble, we need to include these species in the simulation ensemble. Although the Replica exchange method can effectively enhance the sampling efficiency, a single replica may still not contain some rare events, such as the complete completely unfolded state at low temperature or the native state at high temperature (**Fig. S9**). To this end, we utilize the Multistate Bennett Acceptance Ratio (MBAR)[9] method to reweight/merge all the replicas to one ensemble at a specific temperature. Any statistical properties $\hat{A}$ can be estimated by

$$\hat{A} = \sum_{j=1}^{K} \sum_{i=1}^{N_j} A(x_i^j)\rho_i^j \quad \text{(eq. S17)}$$

where j is the replica index, K is the number of replica, i is the frame index, N_j is the number of frame in replica j, x_i^j is the conformation, and ρ_i^j is the corresponding probability from MBAR. In this way, all species that appear in different replicas can be included in one ensemble (**Fig. S10**).

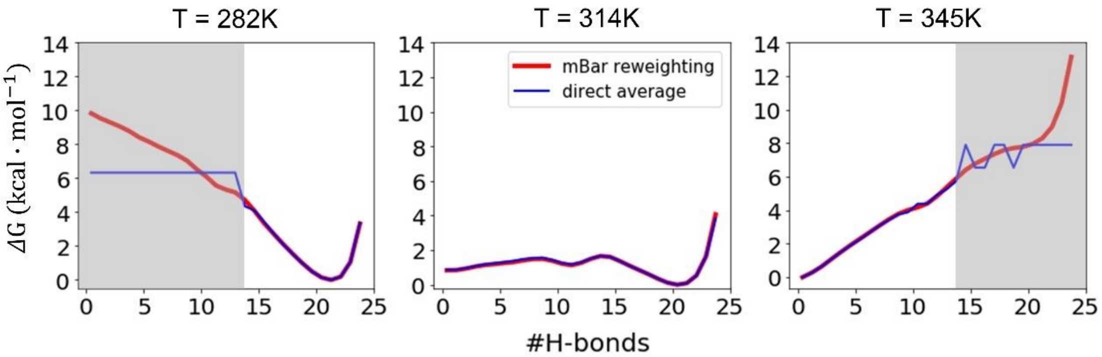

Fig S10. Replica exchange and MBAR renormalization. *Upside* trajectories are run at various temperatures using Replica Exchange protocols and merged using the MBAR procedure[8] to obtain a more accurate ensemble and FES under native conditions. The gray area represents the region where the direct sampling is inadequate and the FES and HX cannot be estimated reliably without MBAR renormalization.

The model for HDX and its denaturant dependence

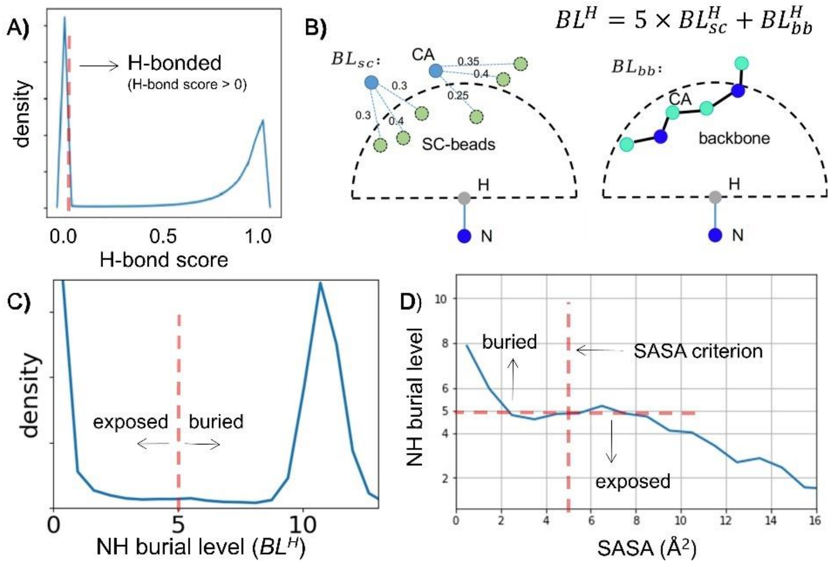

Fig S11. Identifying exchange competent NHs. **A)** H-bond scores from an *Upside* simulation at the T_m are bimodal (near 0 or 1). **B)** Total NH burial level (BL^H) is determined by the side chain beads and backbone atoms. A scale factor of 5 is used to increase the side chain component relative to the backbone component as the side chain bead is a single interaction center and hence under-represents the volume occupied by a true side chain. **C)** A typical distribution of *BL* from *Upside* simulation (residue R13 in EHEE_rd2_0005). A BL^H level of 5 is selected to define an NH as exposed (this value of five and the one in panel B are coincidental). **D)** The relationship between SASA of NHs and the BL^H.

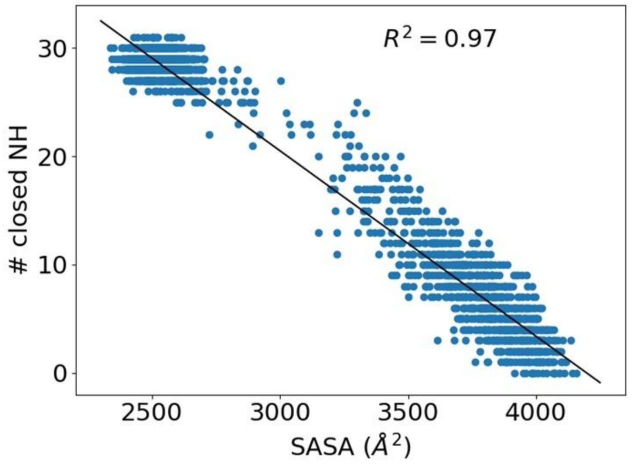

Fig S12. The number of exchange-incompetent NHs (closed) correlates with solvent accessible surface area (SASA) for EHEE_rd2_0005 trajectories.

HDX data fitting

For each protein, we first selected the most stable residues (**Table S3**) as the globally exchanging residues to fit a shared slope with independent intercepts using the least squares method. The shared slope value is assumed to be $\mathrm{m_{global}}$ and the largest intercept is taken to be $\Delta\mathrm{G_{global}}$. (**Table S4**).

Then, a two-state model was used to fit the experimental denaturant dependence of HDX

$$\Delta G_{obs} = -\mathrm{RT}\ln\left(e^{\frac{-(\Delta G_{global}+m_{global}[den])}{RT}} + e^{\frac{-(\Delta G_{subglobal}+m_{subglobal}[den])}{RT}}\right) \quad \text{(eq. S18)}$$

where the ΔG_{global} and m_{global} are shared by all residues. Each residue is given its own $\Delta G_{subglobal}$ and $m_{subglobal}$ which has different slopes from local to sub-global. The slope of the fitted function is used as the "Exp" m-value to compare with the simulated results. ΔG_{global} and m_{global} are used to select the temperature and calibrate the scale factor (s value) for the denaturant sensitivity in our model (**Table S4**).

Table S4. Determine the experimental and simulated ΔG_{global} *and* m_{global}.

protein	the residues used to get m_{global}	ΔG_{global} (kcal·mol^{-1})	m_{global} (kcal·mol^{-1}·M^{-1})	s value (FF2)	s value (FF1)
EHEE_rd2_0005	A12,R13	8.574	0.607	0.038	0.093
HEEH_rd4_0097	E4,R7,R8,L9,E10,V35,E37,E38	4.320	0.871	0.068	0.096
Ub	L15, K27	8.556	1.270	0.051	-
L50E	T7,I13,V17,V26	3.459	1.229	0.061	-

The FES and HDX prediction for designed mini proteins

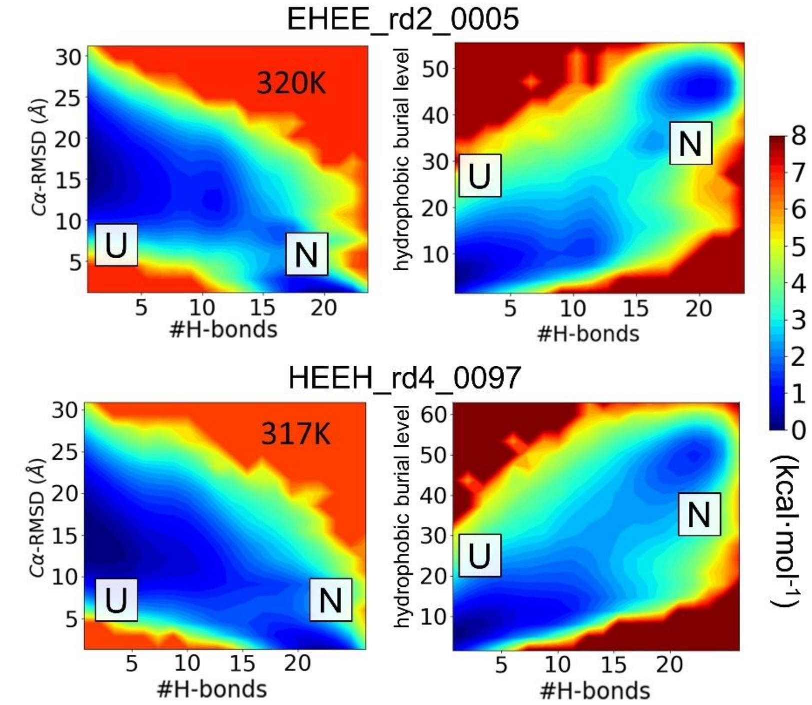

Fig S13. **FES of EHEE_rd2_0005 and HEEH_rd4_0097 variant at T_m** using the number of H-bonds and the Cα-RMSD or burial level as the coordinates.

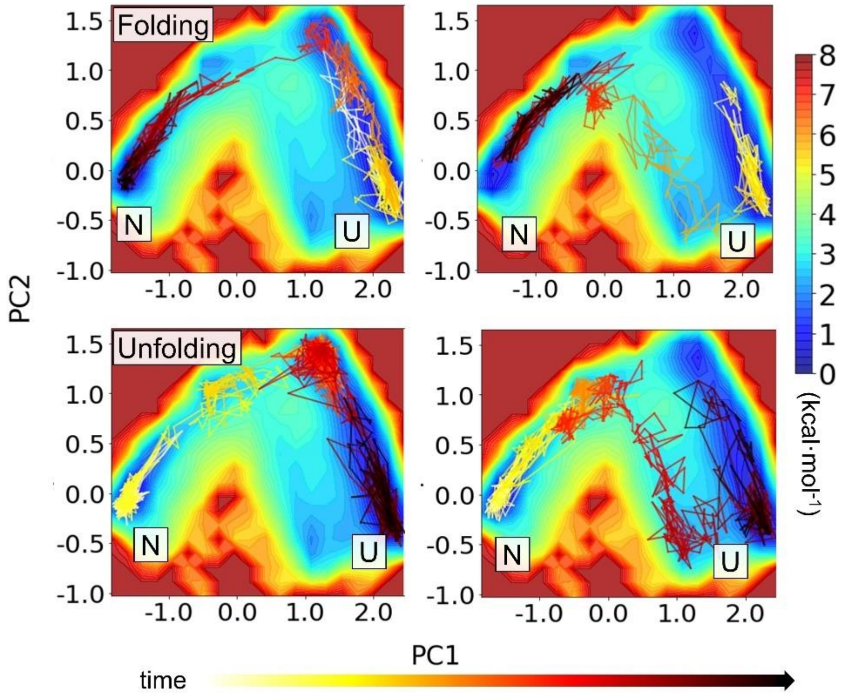

Fig S14. Reversible folding pathways of EHEE_rd2_0005. These pathways are selected from the continuous REMD trajectories across multiple temperatures. EHEE_rd2_0005 reversibly folds along two major pathways.

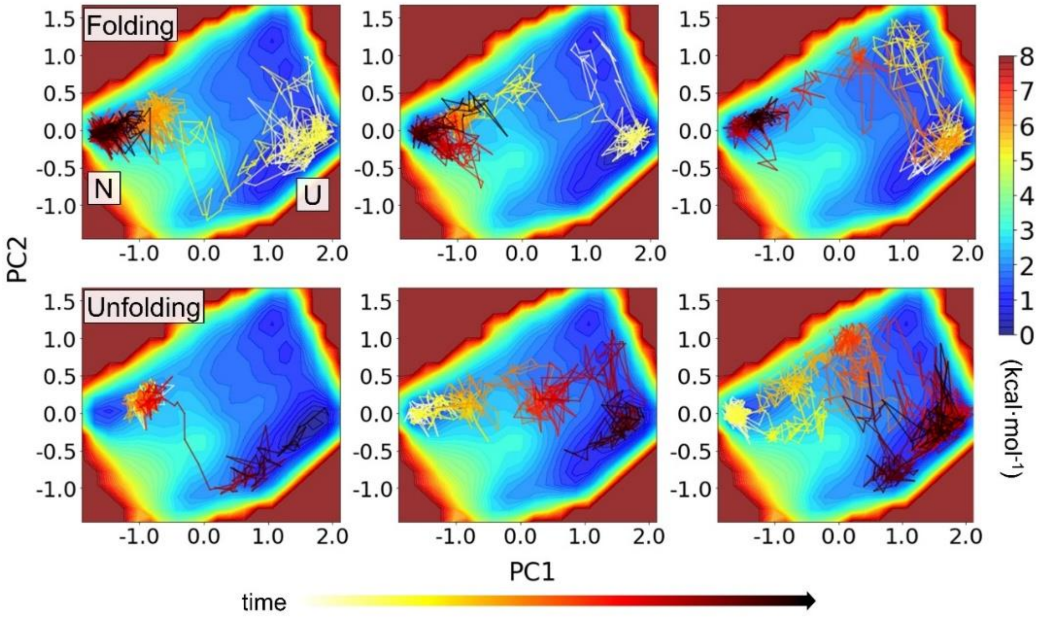

Fig S15. Reversible folding pathways of HEEH_rd4_0097. These pathways are selected from the continuous REMD trajectories (across multiple temperatures). Compared with EHEE_rd2_0005, HEEH_rd4_0097 folds with more pathway diversity.

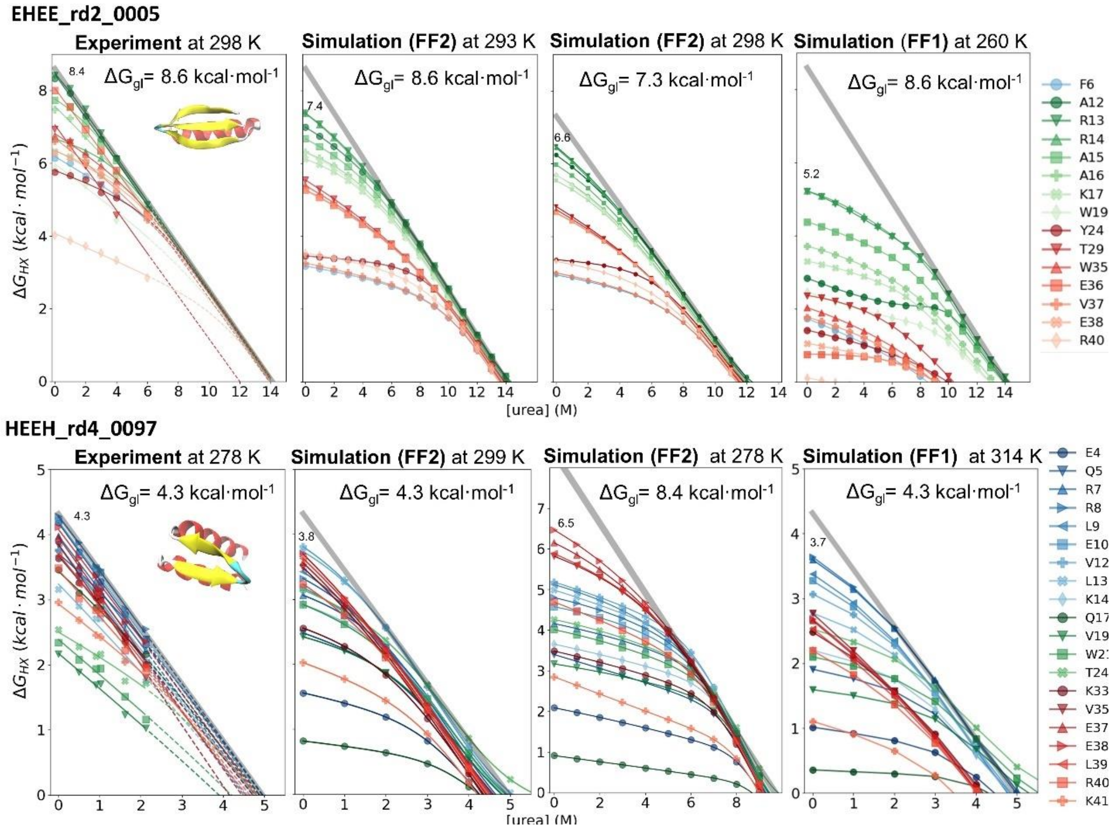

Fig S16. Experimental and simulated HDX patterns for EHEE_rd2_0005 and HEEH_rd4_0097. The global stability and the stability for the most protected NH is shown.

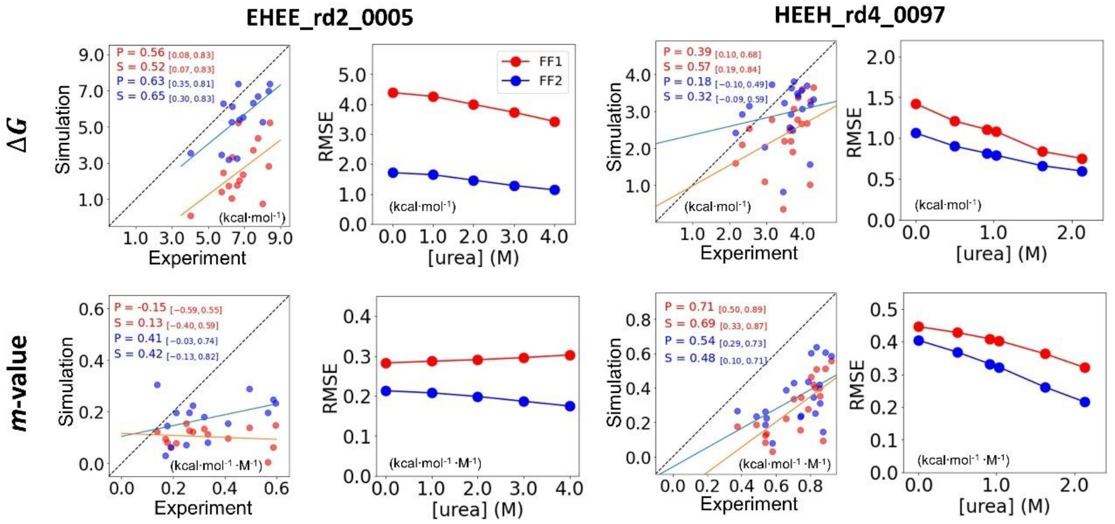

Fig S17. Comparison of FF1 and FF2 about HDX predictions on EHEE_rd2_0005 and HEEH_rd4_0097 in the form of correlation coefficient and Root Mean Square Error (RMSE) to the experimental results.

The simulation results for Ub and L50E

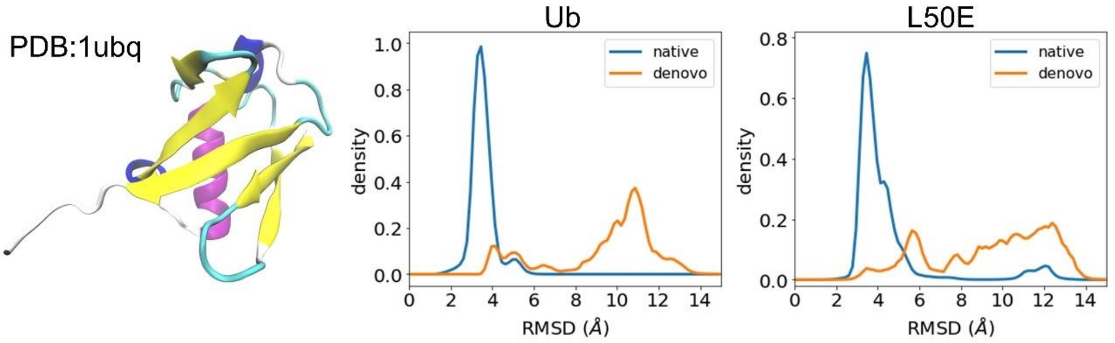

Fig S18. Cα-RMSD distribution of Ub and its L50E variant. Trajectories that begin from the native state largely remain in the native well whereas simulations that start from the unfolded state can populate non-native structures.

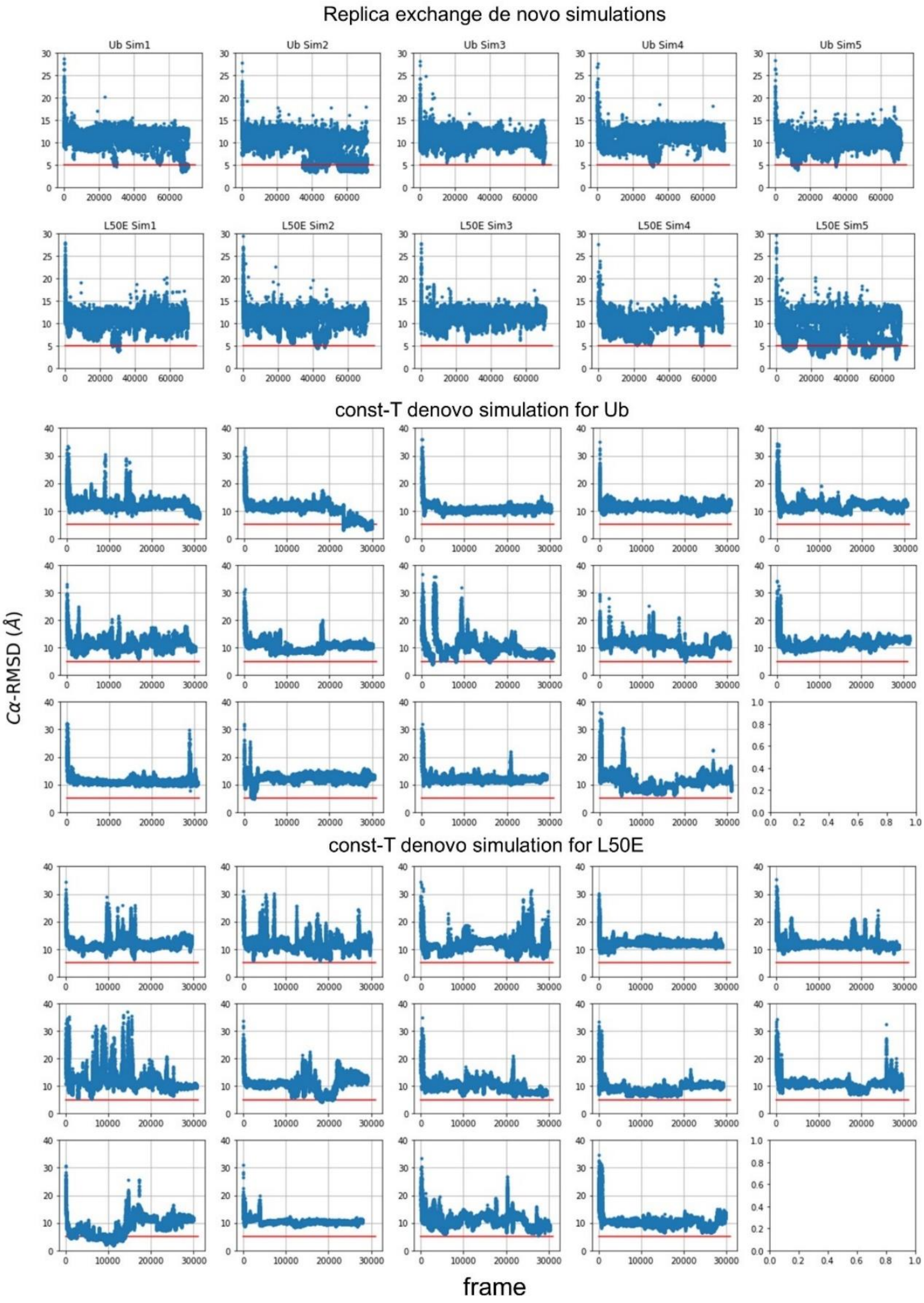

Fig S19. Both de novo REMD and constant temperature MD can find a structure with Cα-RMSD < 5 Å for the Ub and L50E variant.

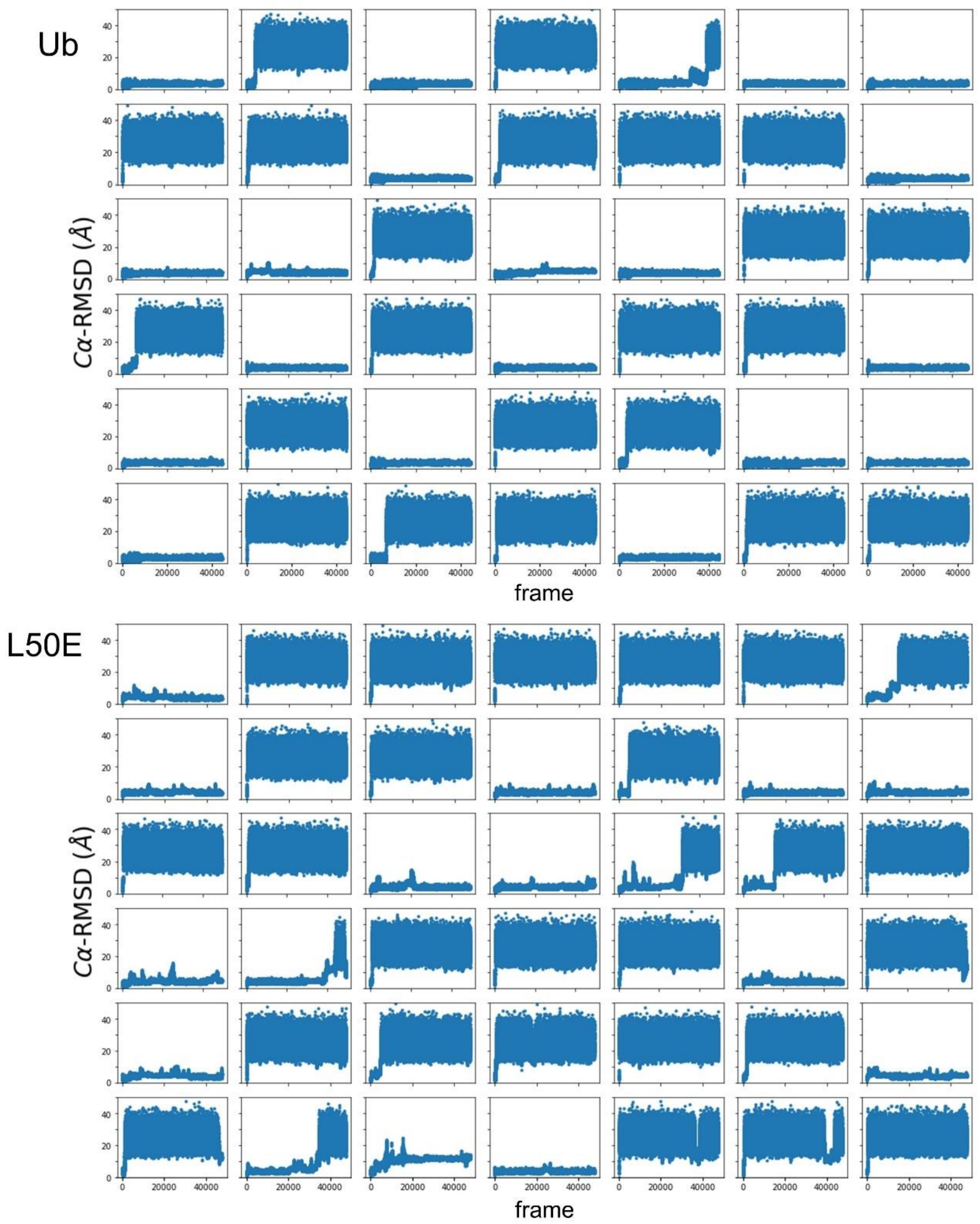

Fig S20. T-REMD simulations for Ub and L50E starting from the native state do not exhibit reversible refolding.

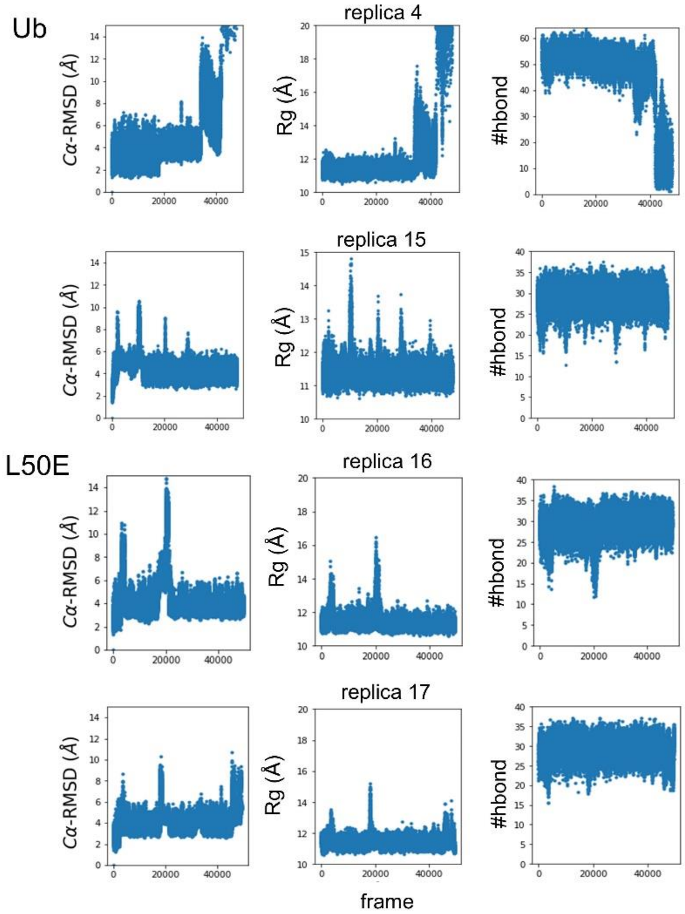

Fig S21. Examples of reversible partial unfolding with Ub and L50E prior to complete unfolding. The trajectories provide structures for calculating the HDX patterns presented in Fig. 9.

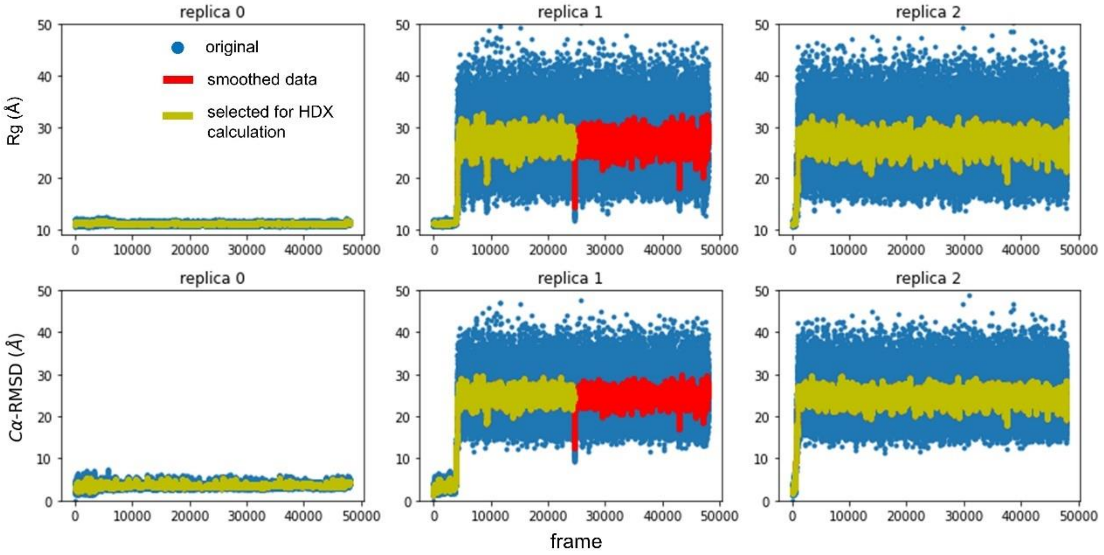

Fig S22. Portion of the trajectory used for HDX calculation when folding is irreversible. For Ub and L50E, only those frames (yellow curve) that are either not fully unfolded or not misfolded after unfolding are selected for determining the FES and HDX behavior. In the smoothed Rg trajectories (red curve), the subsequent frames are discarded when the R_g of an unfolded state is lower than 18 Å.

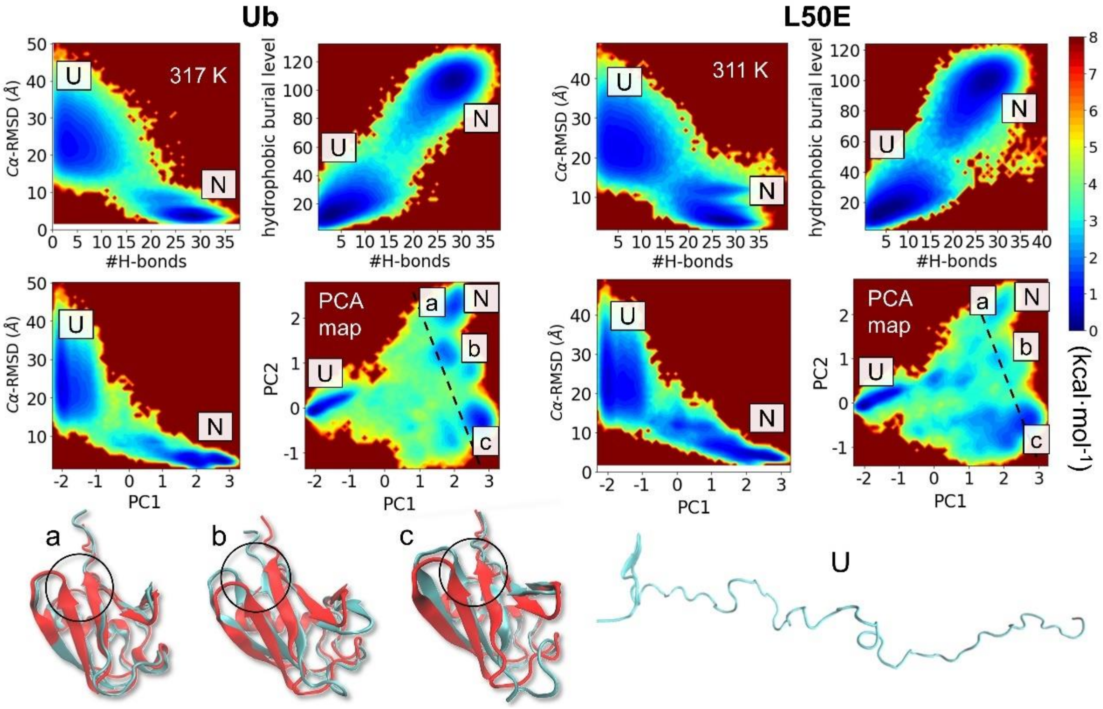

Fig S23. FES of Ub and L50E variant at T_m. These representative structures in NSE and the randomly selected unfolded states (cyan) are shown at the bottom and compared with the native structure (red). The a, b, and c states largely differ in the registry between the amino (β2) and carboxy (β3) terminal strands. In PCA map (lower right), the black dotted line is the NSE boundary.

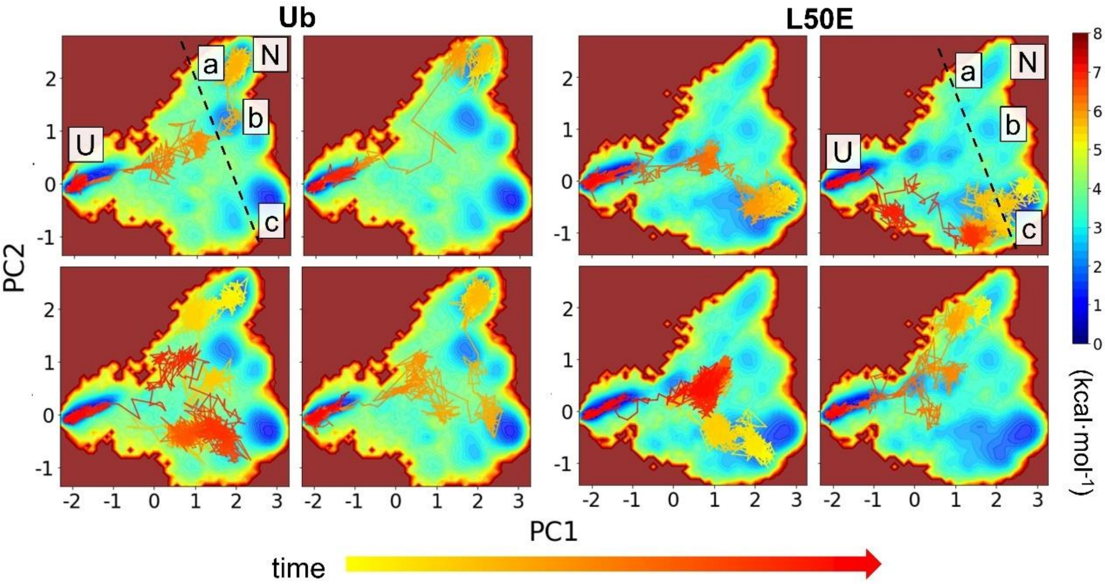

Fig S24. The FES and unfolding pathways of Ub and L50E. These pathways are selected from the continuous REMD trajectories across different temperatures. For both Ub and L50E, there are multiple unfolding pathways. The black dotted line is the NSE boundary.

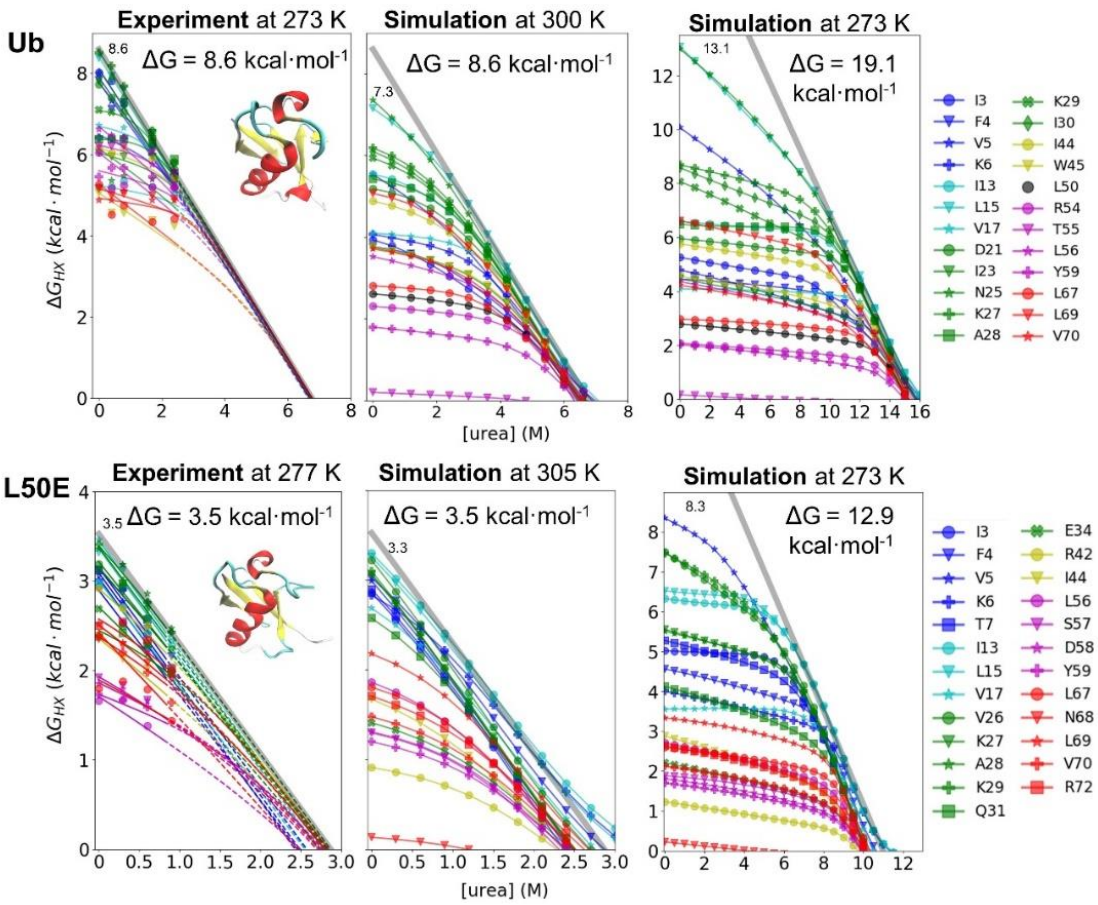

Fig S25. Experimental and simulated HDX patterns for Ub and L50E mutant. The stability value for the most protected NH is provided.

Analysis for local opening

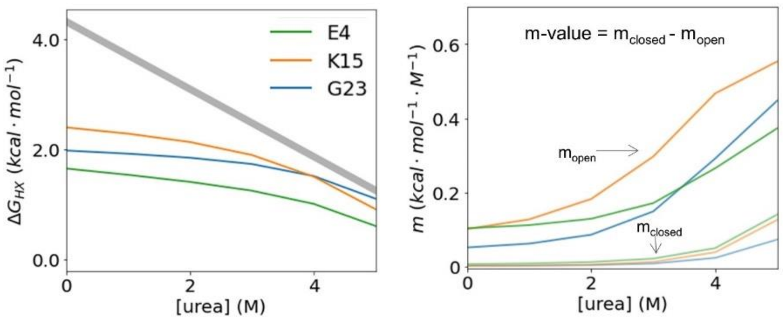

Fig S26. **Local opening events of HEEH_rd4_0097 at T=299 K**. **A)** HDX for 3 residues that exchange by local or near-local openings. **B)** Their m-values are decomposed into values for the closed and open state (m_{closed} and m_{open}).

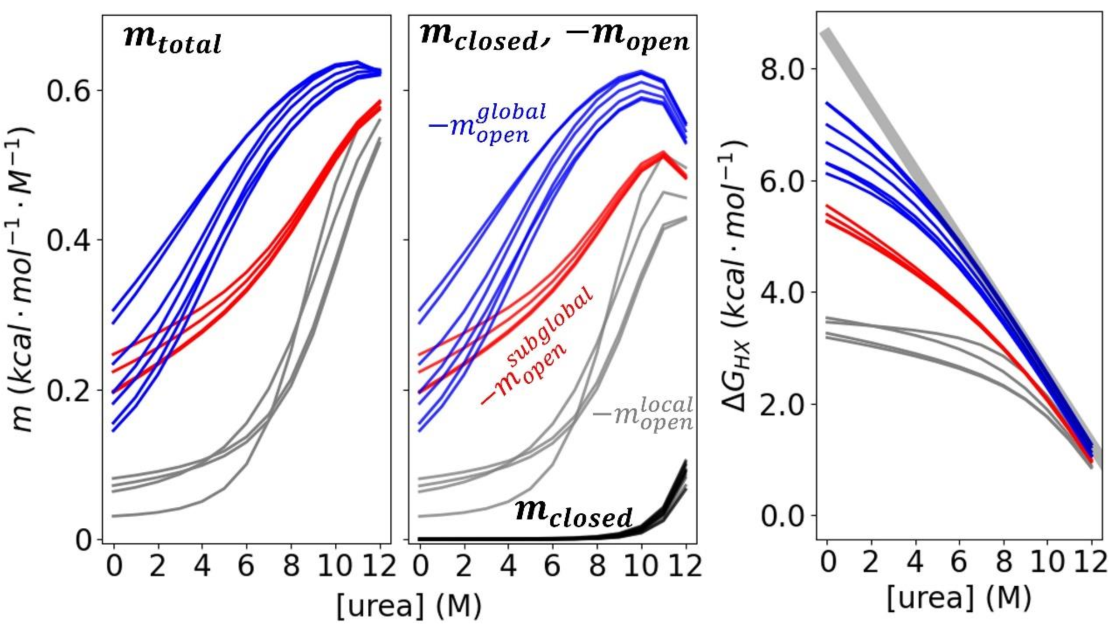

Fig. S27. The m-value decomposition demonstrates that small m-values represent the local breaking of 1-2 H-bonds. The m-values of EHEE_rd2_0005 are decomposed in terms of the H-bonds formed in the closed and open states, i.e. $m_{total}= m_{closed}-m_{open}$. The m-value for the NSE, m_{closed}, is nearly zero across a wide denaturant range, and only increases at very high urea, while m_{open} for each residue (and by consequence m_{total}) is classified according to the associated structural opening: local (gray), subglobal (red), or global (blue). As there is no global residue under stable conditions, residues marked as global are those that first become global exchangers as the denaturant concentration increases. The corresponding free energy dependence on the denaturant is presented on the right.

Experimental methods

Proteins sequences and expression. Proteins were expressed and purified as reported elsewhere [7,10]. The sequences for each protein are:

>EHEE_rd2_0005:

MGSSHHHHHHSSGLVPRGSHM<u>TTRYRFTDEEEARRAAKEWARRGYQVHVTQNGTYWEVEVR</u>

>HEEH_rd4_0097:

GSSHHHHHHSSGLVPRGSHM<u>DVEEQIRRLEEVLKKNQPVTWNGTTYTDPNEIKKVIEELRKSM</u>

>Ub psWT:

MQIFVKTLTGKTITLEVEPSDTIENVKAKIQDKEGIPPDQQRLIWAGKQLEDGRTLSDYNIQKESTLNLVLRLRGG

>Ub L50E:

MQIFVKTLTGKTITLEVEPSDTIENVKAKIQDKEGIPPDQQRLIWAGKQEEDGRTLSDYNIQKESTLNLVLRLRGG

The sequences of EHEE_rd2_0005 and HEEH_rd4_0097 are His-tagged with a thrombin cleavage site. The original underlined portion of the sequence is used for simulation and all related analysis or comparison.

HDX/NMR measurements. HDX was initiated by diluting 100 ul of protein five-fold into the

appropriate D_2O buffer. Deuterated urea was prepared by 3 rounds of dissolving urea and D_2O at a 1:1 w/v ratio followed by lyophilization.

Ub psWT (Ub F45W/H68N)*:* Spectra were collected on a Varian Inova 500 spectrometer at 273 K in 15mM HEPES, 225mM NaCl in pD_{read} 7.6. 2048 complex points were obtained in the ^1H dimension, and 128 complex points were collected in the ^{15}N dimension. Peak assignments were taken from ref [10]. Data was processed using NMRPipe, and peak volumes were measured in VnmrJ.

Ub L50E (Ub F45W/H68N/L50E): Data are obtained from ref [10]. The final HX buffer for UbL50E was 15mM sodium phosphate, 225mM NaCl, 10mM Glu, 10mM Arg, pD_{read} 7.5.

EHEE_rd2_0005, *HEEH_rd4_0097:* HX buffers for EHEE_rd2_0005 and HEEH_rd4_0097 were 20mM sodium phosphate 150mM NaCl pD_{read} 7.1 and 20mM sodium acetate 150mM NaCl pD_{read} 4.6, respectively. NMR spectra were acquired on a Bruker AVANCE III 500MHz NMR spectrometer equipped with a room temperature TXI probe with Topspin 3. A series of ^1H-^{15}N HSQC or BEST-HSQC experiments[11,12] were acquired as a function of time after initiating HX at 298 K (EHEE_rd2_0005) or 278 K (HEEH_rd4_0097). 16 scans were collected per increment, for a total spectral acquisition time of 1.7 hours. In buffer/denaturant conditions where relative exchange rates were rapid and shorter acquisition times were required for accurate HX rate determinations, we used BEST-HSQC. The only parameters changed were the D1 recycle delay was shortened to 0.1 s, and each directly detected FID was collected with only 512 complex points decreasing the acquisition time to 73 ms. The total spectral acquisition time was decreased to 20 minutes. All data for EHEE_rd2_0005 and HEEH_rd4_0097 were processed in NMRFx Analyst[13].

Exchange rates were determined by fitting peak volumes to a single exponential function,

$$y(t) = Ae^{-k_{HX}\cdot t} + y_0,$$

where k_{HX} is the exchange rate, A is the amplitude at zero time and y_0 is the baseline offset (reflecting the 80% D_2O buffer). In series for in HEEH_rd4_0097 where complete exchange of all amides was never reached, kinetic traces across multiple denaturant concentrations were fit simultaneously in OriginPro[14] using shared y_0 while A and k_{HX} were allowed to vary.

Kinetic studies. Refolding data for EHEE_rd2_0005 were collected in 50 mM HEPES, 150mM NaCl, pH 7.54 at 298 K using a Biologic SFM-4000 stopped-flow apparatus monitoring tryptophan fluorescence with a PTI A101 arc lamp as described in ref[15].

Table S5. The $\Delta G_{HX,\mathrm{Exp}}$ for EHEE_rd2_0005 (kcal·mol^{-1}).

seq	0 M	1 M	2 M	3 M	4 M	6 M
F6	6.141±0.023	6.002±0.028	5.649±0.141	5.384±0.074	5.108±0.630	-
A12	8.329±0.009	7.923±0.008	7.293±0.026	6.667±0.013	6.138±0.040	4.742±0.144
R13	8.408±0.013	8.051±0.009	7.486±0.038	6.828±0.021	6.114±0.072	4.851±0.531
R14	6.661±0.019	6.561±0.020	6.313±0.086	6.131±0.035	5.784±0.141	-
A15	7.729±0.007	7.532±0.008	7.112±0.036	6.551±0.021	6.080±0.071	-
A16	7.468±0.006	7.235±0.008	6.754±0.030	6.281±0.021	5.666±0.075	-
K17	6.318+0.015	6.196+0.017	6.036+0.065	5.635+0.041	5.522+0.138	-
W19	5.844±0.014	5.724±0.017	5.404±0.053	5.242±0.019	4.434±0.188	-
Y24	5.750±0.049	5.662±0.043	5.551±0.199	5.195±0.104	-	-
T29	6.931±0.009	6.560±0.012	5.917±0.061	5.407±0.048	4.569±0.945	-
W35	6.744±0.015	6.624±0.018	6.251±0.067	5.915±0.032	5.510±0.088	-
E36	8.000±0.014	7.547±0.013	6.923±0.051	6.343±0.021	5.792±0.090	4.593±0.275
V37	6.603±0.012	6.432±0.012	6.058±0.045	5.675±0.025	5.215±0.069	4.441±0.105
E38	6.278±0.012	6.140±0.014	5.982±0.055	5.767±0.023	5.340±0.084	4.542±0.221
R40	4.022±0.013	3.867±0.015	3.718±0.051	3.530±0.020	3.329±0.071	2.849±0.083

Table S6. The $\Delta G_{HX,\mathrm{Exp}}$ for HEEH_rd4_0097 (kcal·mol^{-1}).

seq	0 M	0.5 M	0.914 M	1.03 M	1.62 M	2.13 M
E4	4.182±0.005	3.731±0.006	3.389±0.006	3.281±0.006	2.775±0.009	2.309±0.013
Q5	3.722±0.006	3.401±0.006	3.177±0.010	3.123±0.010	2.613±0.015	2.394±0.017
R7	3.971±0.008	3.495±0.010	3.294±0.007	3.230±0.007	2.531±0.010	2.219±0.018
R8	4.277±0.006	3.871±0.008	3.509±0.007	3.456±0.008	2.859±0.011	2.551±0.014
L9	3.870±0.009	3.330±0.011	3.106±0.007	3.043±0.009	2.410±0.010	2.038±0.013
E10	4.208±0.010	3.693±0.011	3.481±0.007	3.421±0.009	2.746±0.011	2.376±0.014
V12	3.753±0.009	3.384±0.010	3.186±0.006	3.156±0.011	2.508±0.009	2.044±0.011
L13	3.155±0.007	2.900±0.008	2.706±0.005	2.708±0.006	2.070±0.007	1.750±0.008
K14	3.661±0.008	3.285±0.010	3.058±0.006	3.028±0.007	2.450±0.009	2.034±0.012
Q17	3.460±0.015	3.106±0.019	2.884±0.012	2.857±0.013	2.355±0.017	2.161±0.018
V19	2.162±0.009	1.884±0.012	1.685±0.006	1.702±0.010	1.232±0.009	1.025±0.011
W21	2.342±0.075	2.076±0.092	1.936±0.057	1.943±0.056	1.510±0.068	1.157±0.082
T24	2.536±0.025	2.277±0.030	2.126±0.021	2.162±0.018	1.748±0.024	1.713±0.024
K33	3.636±0.008	3.259±0.010	3.033±0.006	3.003±0.007	2.424±0.009	2.008±0.012
V35	3.843±0.022	3.360±0.023	3.081±0.013	2.988±0.023	2.311±0.023	1.802±0.031
E37	3.954±0.007	3.523±0.009	3.189±0.007	3.119±0.008	2.444±0.012	2.027±0.015
E38	4.113±0.005	3.633±0.006	3.375±0.006	3.152±0.007	2.661±0.011	2.355±0.013
L39	3.678±0.010	3.230±0.013	3.068±0.010	2.974±0.010	2.326±0.011	1.963±0.015
R40	3.486±0.008	3.015±0.020	2.838±0.006	2.795±0.007	2.215±0.007	1.956±0.009
K41	2.959±0.018	2.685±0.022	2.456±0.014	2.429±0.014	2.041±0.018	1.884±0.020

Table S7. The $\Delta G_{HX,\mathrm{Exp}}$ for Ub (kcal·mol^{-1}).

Seq	0 M	0.4 M	0.8 M	1.7 M	2.4 M
I3	8.02	7.82	7.28	6.25	5.57
F4	7.84	7.13	7.29	6.13	5.43
V5	8	7.32	7.17	6.32	5.44
K6	6.42	6.4	6.4	6.01	5.51
I13	5.2	5.2	5.35	5.2	5.08
L15	8.38	7.94	7.61	6.52	5.76
V17	6.72	6.41	6.67	6.37	5.85
D21	7.73	7.41	7.24	6.24	5.64
I23	6.05	5.96	5.96	5.95	5.35
N25	6.16	5.36	5.47	5.39	5.31
K27	8.52	8.19	7.69	6.59	5.79
A28	6.38	6.38	6.38	6.38	5.89
K29	7.1	7.05	7.04	6.56	5.81
I30	7.85	7.89	7.4	6.35	5.47
I44	6.16	6.04	6.16	5.9	5.44
W45	5.06	4.56	4.56	4.38	4.24
L50	5.27	4.21	-	-	-
R54	6.05	5.17	5.24	5.2	5.2
T55	6.21	6.21	6.21	6.05	5.55
L56	6.62	6.48	6.41	5.76	5.27
Y59	5.45	5.67	5.67	5.45	5.18
L67	5.27	4.52	4.75	4.35	4.42
L69	5.16	4.99	5.04	4.99	4.9
V70	4.88	4.88	5.1	4.88	4.88

Table S8. The $\Delta G_{HX,Sim}$ for EHEE_rd2_0005 (kcal·mol^{-1}).

seq	0 M	1 M	2 M	3 M	4 M	6 M
F6	3.179±0.211	3.113±0.206	3.035±0.201	2.950±0.196	2.858±0.190	2.639±0.169
A12	6.992±0.273	6.771±0.235	6.493±0.200	6.174±0.171	5.806±0.147	4.910±0.114
R13	7.378±0.167	7.105±0.159	6.764±0.150	6.380±0.141	5.952±0.131	4.968±0.111
R14	7.375±0.210	7.087±0.186	6.731±0.166	6.336±0.149	5.900±0.134	4.910±0.112
A15	6.670±0.322	6.482±0.271	6.237±0.226	5.943±0.190	5.594±0.161	4.722±0.123
A16	6.304±0.383	6.153±0.313	5.949±0.252	5.697±0.204	5.386±0.169	4.577±0.129
K17	6.115±0.334	5.974±0.280	5.784±0.232	5.547±0.194	5.255±0.167	4.487±0.134
W19	6.295±0.333	6.121±0.277	5.894±0.228	5.622±0.189	5.300±0.161	4.493±0.130
Y24	3.456±0.071	3.428±0.071	3.394±0.071	3.357±0.070	3.313±0.070	3.180±0.083
T29	5.540±0.285	5.313±0.273	5.048±0.260	4.768±0.245	4.471±0.229	3.817±0.191
W35	5.386±0.237	5.180±0.235	4.936±0.231	4.675±0.224	4.396±0.215	3.770±0.185
E36	5.269±0.290	5.085±0.280	4.863±0.270	4.622±0.257	4.359±0.242	3.759±0.203
V37	3.257±0.218	3.182±0.214	3.096±0.210	3.003±0.205	2.904±0.198	2.672±0.175
E38	5.254±0.257	5.072±0.252	4.852±0.246	4.613±0.238	4.352±0.227	3.758±0.195
R40	3.532±0.087	3.473±0.096	3.402±0.107	3.322±0.119	3.230±0.132	2.989±0.150

Table S9. The $\Delta G_{HX,Sim}$ for HEEH_rd4_0097 (kcal·mol^{-1}).

seq	0 M	0.5 M	0.914 M	1.03 M	1.62 M	2.13 M
E4	3.179±0.211	3.113±0.206	3.035±0.201	2.950±0.196	2.858±0.190	2.639±0.169
Q5	6.992±0.273	6.771±0.235	6.493±0.200	6.174±0.171	5.806±0.147	4.910±0.114
R7	7.378±0.167	7.105±0.159	6.764±0.150	6.380±0.141	5.952±0.131	4.968±0.111
R8	7.375±0.210	7.087±0.186	6.731±0.166	6.336±0.149	5.900±0.134	4.910±0.112
L9	6.670±0.322	6.482±0.271	6.237±0.226	5.943±0.190	5.594±0.161	4.722±0.123
E10	6.304±0.383	6.153±0.313	5.949±0.252	5.697±0.204	5.386±0.169	4.577±0.129
V12	6.115±0.334	5.974±0.280	5.784±0.232	5.547±0.194	5.255±0.167	4.487±0.134
L13	6.295±0.333	6.121±0.277	5.894±0.228	5.622±0.189	5.300±0.161	4.493±0.130
K14	3.456±0.071	3.428±0.071	3.394±0.071	3.357±0.070	3.313±0.070	3.180±0.083
Q17	5.540±0.285	5.313±0.273	5.048±0.260	4.768±0.245	4.471±0.229	3.817±0.191
V19	5.386±0.237	5.180±0.235	4.936±0.231	4.675±0.224	4.396±0.215	3.770±0.185
W21	5.269±0.290	5.085±0.280	4.863±0.270	4.622±0.257	4.359±0.242	3.759±0.203
T24	3.257±0.218	3.182±0.214	3.096±0.210	3.003±0.205	2.904±0.198	2.672±0.175
K33	5.254±0.257	5.072±0.252	4.852±0.246	4.613±0.238	4.352±0.227	3.758±0.195
V35	3.532±0.087	3.473±0.096	3.402±0.107	3.322±0.119	3.230±0.132	2.989±0.150
E37	3.179±0.211	3.113±0.206	3.035±0.201	2.950±0.196	2.858±0.190	2.639±0.169
E38	6.992±0.273	6.771±0.235	6.493±0.200	6.174±0.171	5.806±0.147	4.910±0.114
L39	7.378±0.167	7.105±0.159	6.764±0.150	6.380±0.141	5.952±0.131	4.968±0.111
R40	7.375±0.210	7.087±0.186	6.731±0.166	6.336±0.149	5.900±0.134	4.910±0.112
K41	6.670±0.322	6.482±0.271	6.237±0.226	5.943±0.190	5.594±0.161	4.722±0.123

Table S10. The $\Delta G_{HX,Sim}$ for Ub (kcal·mol^{-1}).

seq	0 M	0.4 M	0.8 M	1.7 M	2.4 M
I3	3.933±1.502	3.841±1.453	3.727±1.376	3.382±1.095	3.049±0.809
F4	3.770±1.123	3.720±1.143	3.663±1.154	3.463±1.096	3.196±0.923
V5	5.534±1.954	5.337±1.867	5.131±1.786	4.613±1.615	4.129±1.463
K6	4.069±1.057	4.013±1.073	3.956±1.089	3.806±1.123	3.638±1.133
I13	5.545±0.794	5.470±0.785	5.366±0.744	5.004±0.530	4.622±0.398
L15	7.157±0.400	6.932±0.308	6.694±0.303	6.079±0.524	5.460±0.680
V17	4.098±0.058	4.076±0.061	4.050±0.066	3.972±0.090	3.859±0.148
D21	5.184±0.787	5.123±0.757	5.043±0.719	4.751±0.582	4.375±0.455
I23	3.749±0.113	3.688±0.127	3.623±0.143	3.458±0.177	3.286±0.191
N25	7.355±0.339	7.070±0.355	6.765±0.415	5.984±0.619	5.260±0.742
K27	6.182±0.382	6.061±0.379	5.910±0.379	5.423±0.412	4.887±0.502
A28	5.426±0.247	5.365±0.263	5.289±0.280	5.016±0.338	4.639±0.433
K29	5.938±0.405	5.821±0.417	5.682±0.426	5.245±0.443	4.750±0.483
I30	6.098±0.510	5.964±0.519	5.806±0.521	5.314±0.498	4.784±0.503
I44	4.888±0.465	4.811±0.458	4.721±0.442	4.439±0.360	4.117±0.332
W45	3.769±0.216	3.703±0.200	3.634±0.183	3.460±0.138	3.293±0.113
L50	2.612±0.084	2.581±0.084	2.550±0.084	2.473±0.084	2.402±0.083
R54	2.307±0.355	2.282±0.348	2.255±0.340	2.190±0.319	2.131±0.298
T55	0.199±0.033	0.186±0.032	0.172±0.032	0.141±0.031	0.117±0.030
L56	3.522±0.163	3.453±0.145	3.381±0.125	3.205±0.074	3.044±0.035
Y59	1.797±0.062	1.765±0.062	1.732±0.062	1.655±0.062	1.589±0.060
L67	2.809±0.999	2.789±0.993	2.768±0.985	2.709±0.962	2.635±0.925
L69	5.071±1.196	5.001±1.164	4.914±1.122	4.602±0.964	4.206±0.773
V70	3.713±0.874	3.658±0.858	3.599±0.839	3.434±0.767	3.231±0.635

Table S11. The $\Delta G_{HX,Sim}$ for L50E (kcal·mol^{-1}).

seq	0 M	0.3 M	0.6 M	0.9 M
I3	2.762±0.619	2.454±0.644	2.123±0.670	1.771±0.692
F4	2.944±0.567	2.681±0.586	2.382±0.608	2.053±0.624
V5	2.888±0.631	2.563±0.658	2.219±0.681	1.857±0.696
K6	2.779±0.433	2.606±0.471	2.387±0.513	2.123±0.549
T7	2.801±0.609	2.495±0.649	2.162±0.679	1.808±0.699
I13	3.206±0.543	2.920±0.514	2.618±0.524	2.298±0.551
L15	3.161±0.558	2.866±0.541	2.553±0.556	2.222±0.583
V17	2.625±0.491	2.422±0.519	2.176±0.552	1.891±0.583
V26	3.109±0.835	2.756±0.805	2.388±0.779	2.008±0.755
K27	2.956±0.827	2.624±0.803	2.269±0.783	1.897±0.763
A28	2.954±0.847	2.615±0.821	2.254±0.797	1.878±0.776
K29	2.987±0.897	2.636±0.852	2.269±0.816	1.890±0.787
Q31	2.517±0.630	2.311±0.660	2.059±0.685	1.763±0.701
E34	1.387±0.140	1.298±0.179	1.188±0.231	1.051±0.295
R42	0.900±0.067	0.845±0.080	0.775±0.107	0.682±0.151
I44	1.639±0.106	1.511±0.164	1.358±0.236	1.178±0.315
L56	1.829±0.430	1.708±0.457	1.554±0.487	1.363±0.518
S57	1.279±0.341	1.205±0.362	1.108±0.391	0.980±0.429
D58	1.292±0.291	1.218±0.316	1.123±0.350	0.999±0.392
Y59	1.182±0.183	1.115±0.210	1.029±0.249	0.917±0.299
L67	1.776±0.647	1.674±0.645	1.539±0.645	1.362±0.647
N68	0.132±0.014	0.107±0.026	0.077±0.044	0.036±0.070
L69	2.141±0.615	2.014±0.619	1.849±0.627	1.639±0.637
V70	1.455±0.235	1.370±0.262	1.262±0.300	1.126±0.348
R72	1.678±0.253	1.576±0.286	1.449±0.329	1.292±0.378

Table S12 NMR Peak Assignments for EHEE_rd2_0005 collected at pH 7.56 300K

Assignment	w1 (ppm)	w2 (ppm)
L14N-HN	121.531	7.976
V15N-HN	122.435	7.997
R17N-HN	122.086	8.411
T23N-HN	120.556	8.397
R24N-HN	123.702	8.012
Y25N-HN	123.507	8.909
R26N-HN	122.233	8.445
F27N-HN	121.603	9.423
T28N-HN	112.126	9.075
D29N-HN	120.636	7.621
E31N-HN	117.659	7.99
E32N-HN	120.085	7.889
A33N-HN	121.548	7.22
R34N-HN	115.808	8.215
R35N-HN	121.394	8.014
A36N-HN	123.065	7.656
A37N-HN	119.434	8.453
K38N-HN	117.542	7.692
E39N-HN	120.448	7.514
W40N-HN	119.228	8.203
A41N-HN	122.869	8.526
R42N-HN	121.605	8.168
R43N-HN	116.577	7.496
G44N-HN	105.927	7.895
Y45N-HN	119.205	7.607
Q46N-HN	121.388	9.085
V47N-HN	119.728	8.311
H48N-HN	122.886	8.559
V49N-HN	127.496	8.667
T50N-HN	120.632	9.189
G53N-HN	113.484	8.622
W56N-HN	121.583	9.148
E57N-HN	122.966	9.535
V58N-HN	124.032	9.349
E59N-HN	128.543	9.087
V60N-HN	126.471	8.71
R61N-HN	131.579	8.885

Table S13 NMR Peak Assignments for HEEH_rd4_0097

	pH 5.0		pH 7.0	
**Assignment**	**w1 (ppm)**	**w2 (ppm)**	**w1 (ppm)**	**w2 (ppm)**
M2N-HN	122.399	8.396	121.818	8.296
D3N-HN	122.432	8.293	122.598	8.266
V4N-HN	122.902	8.098	122.877	8.116
E5N-HN	119.038	8.19	118.939	8.227
E6N-HN	119.54	7.597	119.637	7.582
Q7N-HN	119.662	7.774	119.015	7.779
I8N-HN	118.927	8.155	118.366	8.15
R9N-HN	118.79	7.68	118.495	7.619
R10N-HN	118.625	7.862	118.127	7.873
L11N-HN	120.446	7.772	120.238	7.738
E12N-HN	117.97	8.475	118.199	8.529
E13N-HN	119.722	7.681	119.201	7.628
V14N-HN	118.019	7.331	117.623	7.346
L15N-HN	120.275	7.557	120.27	7.615
K16N-HN	118.779	7.437	118.38	7.445
K17N-HN	116.113	7.333	115.827	7.336
N18N-HN	116.64	7.818	116.338	7.823
Q19N-HN	117.088	7.321	116.825	7.305
V21N-HN	117.549	8.198	117.356	8.189
T22N-HN	124.309	8.592	123.988	8.523
W23N-HN	128.796	9.175	128.454	9.155
G25N-HN	102.931	8.286	102.725	8.266
T26N-HN	118.402	7.473	118.235	7.496
T27N-HN	123.201	8.618	122.885	8.538
Y28N-HN	128.727	9.449	128.445	9.42
T29N-HN	110.925	8.446	110.672	8.445
D30N-HN	123.969	7.771	124.049	7.828
N32N-HN	115.072	8.28	114.79	8.312
E33N-HN	119.148	7.514	118.852	7.522
W33NE1-HE1	129.16	10.008	128.865	9.992
I34N-HN	120.467	7.256	120.019	7.268
K35N-HN	118.509	7.965	118.338	7.928
K36N-HN	118.656	6.923	118.139	6.911
V37N-HN	121.391	7.076	120.932	7.053
I38N-HN	119.073	7.567	118.852	7.522
E39N-HN	117.441	7.675	117.286	7.722
E40N-HN	119.288	7.663	119.041	7.651
L41N-HN	121.692	8.051	121.228	7.996
R42N-HN	119.174	8.254	118.496	8.198

Table S14. The overall agreement of predicted ΔG_{HX} and m-value with experiment. Both Pearson, Spearman correlation coefficient and Root Mean Square Error (RMSE) are listed with the 90% confidence interval (in parentheses) estimated by bootstrap method.

		[urea] (M)	ΔG			m-value		
			Pearson	Spearman	RMSE (kcal·mol^{-1})	Pearson	Spearman	RMSE (kcal·mol^{-1}·M^{-1})
EHEE_rd2_0005	FF1	0.0	0.556$_{[0.076,0.833]}$	0.521$_{[0.061,0.825]}$	4.382$_{[3.819,4.881]}$	-0.153$_{[-0.595,0.549]}$	0.132$_{[-0.370,0.582]}$	0.283$_{[0.204,0.345]}$
		4.0	0.630$_{[0.148,0.806]}$	0.571$_{[0.068,0.820]}$	3.421$_{[2.943,3.812]}$	0.201$_{[-0.266,0.623]}$	0.207$_{[-0.295,0.621]}$	0.302$_{[0.241,0.355]}$
	FF2	0.0	0.631$_{[0.315,0.805]}$	0.650$_{[0.274,0.848]}$	1.709$_{[1.237,2.080]}$	0.407$_{[0.013,0.729]}$	0.421$_{[-0.102,0.825]}$	0.213$_{[0.163,0.253]}$
		4.0	0.575$_{[0.344,0.808]}$	0.689$_{[0.277,0.823]}$	1.136$_{[0.760,1.426]}$	0.636$_{[0.327,0.803]}$	0.600$_{[0.205,0.826]}$	0.175$_{[0.137,0.205]}$
HEEH_rd4_0097	FF1	0.0	0.393$_{[0.101,0.678]}$	0.574$_{[0.190,0.835]}$	1.421$_{[1.053,1.755]}$	0.714$_{[0.496,0.891]}$	0.686$_{[0.333,0.866]}$	0.446$_{[0.402,0.490]}$
		2.13	0.112$_{[-0.207,0.377]}$	0.257$_{[-0.158,0.589]}$	0.748$_{[0.511,0.943]}$	0.617$_{[0.359,0.844]}$	0.663$_{[0.283,0.879]}$	0.332$_{[0.251,0.405]}$
	FF2	0.0	0.185$_{[-0.097,0.506]}$	0.317$_{[-0.079,0.628]}$	1.063$_{[0.672,1.369]}$	0.541$_{[0.297,0.723]}$	0.475$_{[0.106,0.707]}$	0.404$_{[0.346,0.454]}$
		2.13	-0.097$_{[-0.357,0.167]}$	0.036$_{[-0.359,0.416]}$	0.602$_{[0.413,0.756]}$	0.407$_{[0.113,0.692]}$	0.439$_{[0.074,0.685]}$	0.216$_{[0.118,0.282]}$
Ub	FF2	0.0	0.455$_{[0.211,0.669]}$	0.492$_{[0.186,0.696]}$	2.647$_{[2.175,3.081]}$	0.444$_{[0.197,0.689]}$	0.442$_{[0.099,0.666]}$	0.518$_{[0.394,0.618]}$
		2.4	0.304$_{[0.058,0.580]}$	0.430$_{[0.108,0.647]}$	1.786$_{[1.256,2.275]}$	0.427$_{[0.142,0.672]}$	0.481$_{[0.139,0.711]}$	0.487$_{[0.422,0.546]}$
L50E	FF2	0.0	0.712$_{[0.566,0.856]}$	0.761$_{[0.599,0.849]}$	0.766$_{[0.473,0.992]}$	0.749$_{[0.617,0.846]}$	0.666$_{[0.454,0.754]}$	0.359$_{[0.283,0.418]}$
		0.9	0.525$_{[0.318,0.749]}$	0.618$_{[0.347,0.761]}$	0.552$_{[0.327,0.727]}$	0.741$_{[0.604,0.842]}$	0.678$_{[0.487,0.774]}$	0.243$_{[0.175,0.298]}$

References

[1] Jumper, J. M.; Faruk, N. F.; Freed, K. F.; Sosnick, T. R. Accurate Calculation of Side Chain Packing and Free Energy with Applications to Protein Molecular Dynamics. PLoS Comput. Biol. 2018, 14 (12). https://doi.org/10.1371/journal.pcbi.1006342.

[2] Jumper, J. M.; Faruk, N. F.; Freed, K. F.; Sosnick, T. R. Trajectory-Based Training Enables Protein Simulations with Accurate Folding and Boltzmann Ensembles in Cpu-Hours. PLOS Comput. Biol. 2018, 14 (12), e1006578. https://doi.org/10.1371/journal.pcbi.1006578.

[3] Kabsch, W.; Sander, C. Dictionary of Protein Secondary Structure: Pattern Recognition of Hydrogen-bonded and Geometrical Features. Biopolymers 1983, 22 (12), 2577–2637. https://doi.org/10.1002/bip.360221211.

[4] Ester, M., H. P. Kriegel, J. Sander, and X. Xu, "A Density-Based Algorithm for Discovering Clusters in Large Spatial Databases with Noise". In: Proceedings of the 2nd International Conference on Knowledge Discovery and Data Mining, Portland, OR, AAAI Press, pp. 226-231. 1996

[5] Schubert, E., Sander, J., Ester, M., Kriegel, H. P., & Xu, X. (2017). DBSCAN revisited, revisited: why and how you should (still) use DBSCAN. ACM Transactions on Database Systems (TODS), 42(3), 19. https://dl.acm.org/doi/10.1145/3068335

[6] Zhang Y.; Skolnick J. Scoring function for automated assessment of protein structure template quality. Proteins. 2004, 57 (4), 702–710. https://doi.org/10.1002/prot.20264

[7] Rocklin, G. J.; Chidyausiku, T. M.; Goreshnik, I.; Ford, A.; Houliston, S.; Lemak, A.; Carter, L.; Ravichandran, R.; Mulligan, V. K.; Chevalier, A.; Arrowsmith, C. H.; Baker, D. Global Analysis of Protein Folding Using Massively Parallel Design, Synthesis, and Testing. Science (80-.). 2017, 357 (6347), 168 – 175. https://doi.org/10.1126/science.aan0693.

[8] Gillespie, D. T. Exact Numerical Simulation of the Ornstein-Uhlenbeck Process and Its Integral. Phys. Rev. E - Stat. Physics, Plasmas, Fluids, Relat. Interdiscip. Top. 1996, 54 (2), 2084–2091. https://doi.org/10.1103/PhysRevE.54.2084.

[9] Shirts, M. R.; Chodera, J. D. Statistically Optimal Analysis of Samples from Multiple Equilibrium States. J. Chem. Phys. 2008, 129 (12), 124105. https://doi.org/10.1063/1.2978177.

[10] Zheng, Z.; Sosnick, T. R. Protein Vivisection Reveals Elusive Intermediates in Folding. J. Mol. Biol. 2010, 397 (3), 777 – 788. https://doi.org/10.1016/j.jmb.2010.01.056.

[11] Schanda, P.; Van Melckebeke, H.; Brutscher, B. Speeding up Three-Dimensional Protein NMR Experiments to a Few Minutes. J. Am. Chem. Soc. 2006, 128 (28), 9042–9043. https://doi.org/10.1021/ja062025p.

[12] Lescop, E.; Schanda, P.; Brutscher, B. A Set of BEST Triple-Resonance Experiments for Time-Optimized Protein Resonance Assignment. J. Magn. Reson. 2007, 187 (1), 163–169. https://doi.org/10.1016/j.jmr.2007.04.002.

[13] Norris, M.; Fetler, B.; Marchant, J.; Johnson, B. A. NMRFx Processor: A Cross-Platform NMR Data Processing Program. J. Biomol. NMR 2016, 65 (3–4), 205–216. https://doi.org/10.1007/s10858-016-0049-6.

[14] Origin(Pro), Version 2021. OriginLab Corporation, Northampton, MA, USA.

[15] Yu, W.; Baxa, M. C.; Gagnon, I.; Freed, K. F.; Sosnick, T. R. Cooperative Folding near the Downhill Limit Deter-mined with Amino Acid Resolution by Hydrogen Exchange. Proc. Natl. Acad. Sci. U. S. A. 2016, 113 (17), 4747–4752. https://doi.org/10.1073/pnas.1522500113.
